# Spectrum and frequency of genetic variants in sporadic amyotrophic lateral sclerosis

**DOI:** 10.1093/braincomms/fcad152

**Published:** 2023-05-09

**Authors:** Wolfgang P Ruf, Matej Boros, Axel Freischmidt, David Brenner, Veselin Grozdanov, Joao de Meirelles, Thomas Meyer, Torsten Grehl, Susanne Petri, Julian Grosskreutz, Ute Weyen, Rene Guenther, Martin Regensburger, Tim Hagenacker, Jan C Koch, Alexander Emmer, Annekathrin Roediger, Robert Steinbach, Joachim Wolf, Jochen H Weishaupt, Paul Lingor, Marcus Deschauer, Isabell Cordts, Thomas Klopstock, Peter Reilich, Florian Schoeberl, Berthold Schrank, Daniel Zeller, Andreas Hermann, Antje Knehr, Kornelia Günther, Johannes Dorst, Joachim Schuster, Reiner Siebert, Albert C Ludolph, Kathrin Müller

**Affiliations:** Department of Neurology, Ulm University, Ulm 89081, Germany; Institute of Human Genetics, Ulm University & Ulm University Medical Center, Ulm 89081, Germany; Department of Neurology, Ulm University, Ulm 89081, Germany; Deutsches Zentrum für Neurodegenerative Erkrankungen (DZNE), German Center for Neurodegenerative Diseases, Ulm 89081, Germany; Department of Neurology, Ulm University, Ulm 89081, Germany; Department of Neurology, Ulm University, Ulm 89081, Germany; Deutsches Zentrum für Neurodegenerative Erkrankungen (DZNE), German Center for Neurodegenerative Diseases, Ulm 89081, Germany; Department of Neurology, Center for ALS and other Motor Neuron Disorders, Charité—Universitätsmedizin Berlin, Corporate Member of Freie Universität Berlin, Humboldt-Universität zu Berlin, and Berlin Institute of Health, Berlin 13353, Germany; Department of Neurology, Alfried Krupp Hospital, Essen 45131, Germany; Department of Neurology, Medizinische Hochschule Hannover, Hannover 30625, Germany; Precision Neurology, University of Luebeck, Luebeck 23562, Germany; Department of Neurology, University Hospital Bochum, Bochum 44789, Germany; Department of Neurology, Technische Universität Dresden, Dresden 01307, Germany; Department of Neurology, University Hospital Erlangen, Erlangen 91054, Germany; Department of Neurology Center for Translational Neuro- and Behavioral Sciences (C-TNBS), University Medicine Essen, Essen 45147, Germany; Department of Neurology, University Medical Center Goettingen, Goettingen 37075, Germany; University Clinic and Polyclinic for Neurology, University Hospital Halle, Halle 06120, Germany; Department of Neurology, University Hospital Jena, Jena 07747, Germany; Department of Neurology, University Hospital Jena, Jena 07747, Germany; Department of Neurology, Diako Mannheim, Mannheim 68163, Germany; Department of Neurology, University Hospital Mannheim, Mannheim 68167, Germany; Department of Neurology, Technical University Munich, Munich 80333, Germany; Department of Neurology, Technical University Munich, Munich 80333, Germany; Department of Neurology, Technical University Munich, Munich 80333, Germany; Department of Neurology with Friedrich-Baur-Institute, University Hospital of Ludwig-Maximilians-University, München 80336, Germany; Deutsches Zentrum für Neurodegenerative Erkrankungen (DZNE), German Center for Neurodegenerative Diseases, Munich 81377, Germany; Department of Neurology with Friedrich-Baur-Institute, University Hospital of Ludwig-Maximilians-University, München 80336, Germany; Department of Neurology with Friedrich-Baur-Institute, University Hospital of Ludwig-Maximilians-University, München 80336, Germany; Department of Neurology, DKD Helios Clinics, Wiesbaden 65191, Germany; Department of Neurology, University Hospital Wuerzburg, Wuerzburg 97080, Germany; Translational Neurodegeneration Section ‘Albrecht Kossel’, University Medical Center Rostock, Rostock 18146, Germany; Deutsches Zentrum für Neurodegenerative Erkrankungen (DZNE), German Center for Neurodegenerative Diseases, Rostock/Greifswald 17489, Germany; Department of Neurology, Ulm University, Ulm 89081, Germany; Department of Neurology, Ulm University, Ulm 89081, Germany; Department of Neurology, Ulm University, Ulm 89081, Germany; Deutsches Zentrum für Neurodegenerative Erkrankungen (DZNE), German Center for Neurodegenerative Diseases, Ulm 89081, Germany; Department of Neurology, Ulm University, Ulm 89081, Germany; Deutsches Zentrum für Neurodegenerative Erkrankungen (DZNE), German Center for Neurodegenerative Diseases, Ulm 89081, Germany; Institute of Human Genetics, Ulm University & Ulm University Medical Center, Ulm 89081, Germany; Department of Neurology, Ulm University, Ulm 89081, Germany; Deutsches Zentrum für Neurodegenerative Erkrankungen (DZNE), German Center for Neurodegenerative Diseases, Ulm 89081, Germany; Department of Neurology, Ulm University, Ulm 89081, Germany; Institute of Human Genetics, Ulm University & Ulm University Medical Center, Ulm 89081, Germany

**Keywords:** amyotrophic lateral sclerosis, motor neuron disease, genetics

## Abstract

Therapy of motoneuron diseases entered a new phase with the use of intrathecal antisense oligonucleotide therapies treating patients with specific gene mutations predominantly in the context of familial amyotrophic lateral sclerosis. With the majority of cases being sporadic, we conducted a cohort study to describe the mutational landscape of sporadic amyotrophic lateral sclerosis. We analysed genetic variants in amyotrophic lateral sclerosis-associated genes to assess and potentially increase the number of patients eligible for gene-specific therapies. We screened 2340 sporadic amyotrophic lateral sclerosis patients from the German Network for motor neuron diseases for variants in 36 amyotrophic lateral sclerosis-associated genes using targeted next-generation sequencing and for the *C9orf72* hexanucleotide repeat expansion. The genetic analysis could be completed on 2267 patients. Clinical data included age at onset, disease progression rate and survival. In this study, we found 79 likely pathogenic Class 4 variants and 10 pathogenic Class 5 variants (without the *C9orf72* hexanucleotide repeat expansion) according to the American College of Medical Genetics and Genomics guidelines, of which 31 variants are novel. Thus, including *C9orf72* hexanucleotide repeat expansion, Class 4, and Class 5 variants, 296 patients, corresponding to ∼13% of our cohort, could be genetically resolved. We detected 437 variants of unknown significance of which 103 are novel. Corroborating the theory of oligogenic causation in amyotrophic lateral sclerosis, we found a co-occurrence of pathogenic variants in 10 patients (0.4%) with 7 being *C9orf72* hexanucleotide repeat expansion carriers. In a gene-wise survival analysis, we found a higher hazard ratio of 1.47 (95% confidence interval 1.02–2.1) for death from any cause for patients with the *C9orf72* hexanucleotide repeat expansion and a lower hazard ratio of 0.33 (95% confidence interval 0.12–0.9) for patients with pathogenic *SOD1* variants than for patients without a causal gene mutation.

In summary, the high yield of 296 patients (∼13%) harbouring a pathogenic variant and oncoming gene-specific therapies for *SOD1/FUS/C9orf72,* which would apply to 227 patients (∼10%) in this cohort, corroborates that genetic testing should be made available to all sporadic amyotrophic lateral sclerosis patients after respective counselling.

## Teaser

In a study of 2267 sporadic amyotrophic lateral sclerosis patients, Ruf *et al*. report that ∼13% could be genetically resolved with ∼10% being eligible for one of the oncoming gene-specific therapies (SOD1/FUS/C9orf72). They conclude that genetic testing should be made available to all amyotrophic lateral sclerosis patients after respective counselling.

## Graphical Abstract

**Graphical Abstract:**
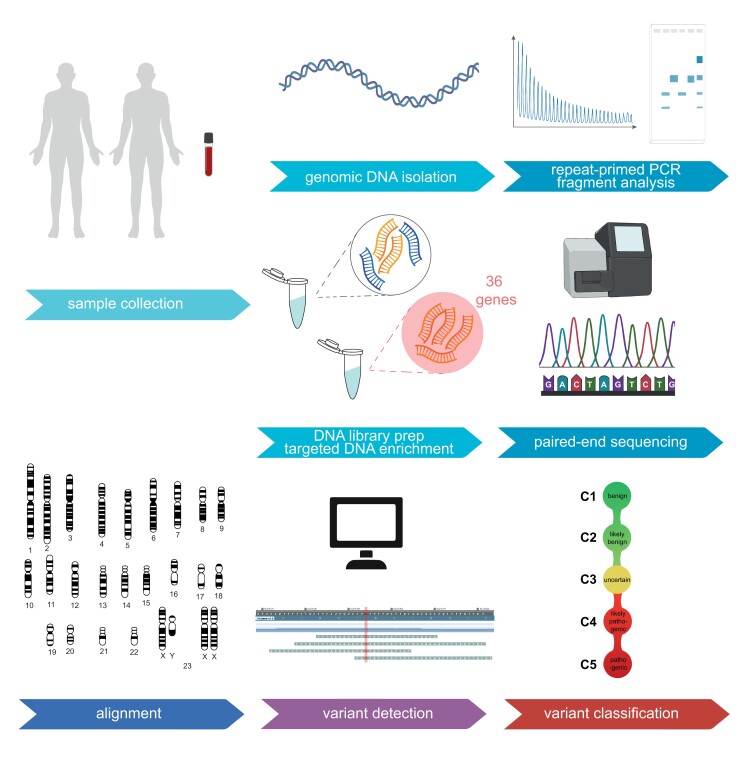

## Introduction

Amyotrophic lateral sclerosis (ALS) is a fatal motoneuron disease (MND) with very limited treatment options.^1^ Many causal therapies target familial ALS (fALS) cases with ALS-associated genetic mutations through intrathecal antisense oligonucleotides. For the most frequently mutated Mendelian ALS genes, antisense oligonucleotide-based therapies are being tested (ION363 for FUS-ALS),^2^ or early access programs are already available (Tofersen for SOD1-ALS).^3^ Despite a setback for the antisense oligonucleotide therapy against the *C9orf72* hexanucleotide repeat expansion (HRE), new clinical trials for variant-specific antisense oligonucleotide therapies for C9orf72-ALS/frontotemporal dementia (FTD) are already recruiting patients (WVE-004 for C9orf72-ALS/FTD). While the overall percentage of pathogenic variants in fALS is very high ranging from 50 to 85%,^4,5^ the reported proportion of pathogenic variants in sporadic amyotrophic lateral sclerosis (sALS) is highly variable depending on the respective study and population, ranging from 7.4% for European sALS to 2.9% for Japanese sALS.^6^ Furthermore, many studies focused on key genes only, such as *C9orf72*, *SOD1*, *TARDBP* and *FUS*, making an overall estimation of ALS-associated variants in sALS difficult. Therefore, obtaining a comprehensive overview of the mutational landscape in a large cohort of sALS in most ALS-associated genes might help to identify more cases eligible for targeted therapy. In addition, a more accurate description of the frequencies of pathogenic variants in ALS genes for which valid frequency distributions in larger cohorts are not yet available may attract further research investments to develop individualized therapies. Hence, we set out to screen 2340 sporadic ALS cases from Germany for pathogenic variants in 36 ALS-associated genes and classify the variants according to the guidelines of the American College of Medical Genetics and Genomics (ACMG), aiming at updating the population statistics of known pathogenic variants and identifying novel variants.

## Materials and methods

### Study design

Patients were recruited in 17 academic referral centres of the German Motor Neuron Disease Network (MND-NET): University Hospital of Bochum, Erlangen, Essen, Göttingen, Halle, Jena, Mannheim, Ulm, Wuerzburg, Berlin Charité, Technical University of Munich, Technische Universität Dresden, Ludwig-Maximilian-University Munich, Alfried Krupp Hospital Essen, Hannover Medical School, Diako Mannheim and DKD Helios Clinics Wiesbaden. ALS patients were diagnosed according to the revised El-Escorial criteria,^7^ primary lateral sclerosis patients were diagnosed according to the criteria of Pringle *et al*.^8^ The diagnosis of sALS and sporadic primary lateral sclerosis was based on the absence of a first- or second-degree relative with ALS/FTD spectrum disorder based on the patient’s or family members’ reporting. Genetic testing was offered to all sALS and sporadic primary lateral sclerosis patients enrolled in the MND-NET project. All patients included provided written informed consent to participate in the genetic studies, which were approved by the local medical ethics committees (lead EC Ulm University, approval no 19/12). In total, 2340 patients could be enrolled in this cohort study from February 2019 to June 2022.

### Clinical data collection

Demographic and clinical patient data, including sex, age at onset, the phenotype of the disease and ALS Functional Rating Scale-Revised (ALSFRS-R) score, were collected during the visit to the respective centre. Disease progression was defined as the rate of decrease in the ALSFRS-R score at enrolment (Δ ALSFRS-R/m) and was calculated as follows: Δ ALSFRS-R/m = (48-ALSFRS-R score at visit)/(date of the visit − date of onset in months). Patients were followed up at subsequent visits to the respective centres collecting current ALSFRS-R scores. For 47 patients, there was a change in the family history for ALS, e.g. newly affected family members (*n* = 38) or a revision of the final diagnosis of ALS (*n* = 9), which were then excluded from the study. Survival time was defined as the interval from symptom onset to the endpoint event or the last follow-up, where death from any cause was defined as an endpoint event. The censoring date for survival data was 1500 days after symptom onset. Patients lost to follow-up were censored at the last known living data point.

### Blood collection

The collection of human peripheral venous blood was performed according to the respective standard operating procedures of each centre.

### Genetic analysis

DNA was extracted from whole ethylenediaminetetraacetic acid-containing venous blood samples.^9^*C9orf72* genotyping in all samples was carried out by fragment analysis and repeat-primed PCR.^10,11^ All normal homozygotes and expanded alleles were confirmed with Southern blot.^12^ The size or length of the DNA was estimated by the marker that was run on the gel along with the DNA sample. The length of each lane containing a different DNA sample was analysed separately based on the migration distance of the DNA containing the GGGGCC-hexanucleotide repeat in *C9orf72* in comparison to the known marker using a semilogarithmic paper. For the targeted gene sequencing, a custom panel from Illumina with 36 genes was used (Supplemental Table 1). The subsequent sequencing was performed for 150 cycles by generating 2× 74 bp paired-end reads on the NextSeq 550 (Illumina) or the MiSeq (Illumina). The coverage of all genes was at least 20×. Enrichment for targeted gene sequencing was performed with the Nextera Rapid Capture Custom Kit (Illumina).

### Variant analysis

Burrows–Wheeler Aligner (0.7.17) with standard parameters was used for reading alignment against the human genome assembly hg19 (GRCh37). We performed single-nucleotide variant (SNV) and small insertion and deletion (indel) variants calling specifically for the targeted regions using SEQNEXT (JSI medical systems) with standard parameters. The variants were lifted over from GRCh37 to GRCh38 with the ENSEMBL tool Assembly converter Ensembl release 106, when necessary for further analysis.^13^ Variants were then further processed with ensembl variant effect predictor (ensemble-vep, version 106.1) and SNPEff version 5.1.^14^ The genomic coordinates of the mutations refer to the GRCh38 genome and were determined using ensemble-vep. The affected part of the transcript was also derived from ensemble-vep. The predicted variant effects in ensemble-vep and SNPEff were combined, and a consensus effect is given in Supplemental Table 1. The ACMG classification criteria were used to classify the variants into benign Class 1 (C1), likely benign Class 2 (C2), variant of uncertain significance, Class 3 (C3), likely pathogenic Class 4 (C4) and pathogenic variants Class 5 (C5).^15^ We extracted the mode of inheritance for different traits of the genes from the Clinical Genomic Database^16^ or Online Mendelian Inheritance in Man.^17^ Whether loss of function (LoF) is a known mechanism of disease for the respective gene was evaluated by multiple sources (Online Mendelian Inheritance in Man), Varsome.com^18^ and by literature research.^19-21^ For the BS1 rule (Allele frequency is greater in databases than expected for the disorder) we used a benign cut-off frequency of 0.0001, except for genes for which pathological variants exist that are more frequent than this limit, e.g. *SOD1.* Here, we used a cut-off frequency corresponding to the known pathogenic variant with the highest allele frequency which is given in Supplemental Table 1. As reference databases, we used Genome Aggregation Database (GnomAD 2.1.1/3.1.2),^22^ Exome Aggregation Consortium (ExAC),^23^ NCBI Allele Frequency Aggregator,^24^ National Heart Lung and Blood Institute Exome Sequencing Project (ESP6500, http://evs.gs.washington.edu/EVS/) [November 2021]), Thousand Genomes Project (TGP),^25^ (UK10K),^26^ The UK Adult Twin Registry (TWINSUK)^27^ and National Heart Lung and Blood Institute Trans-Omics for Precision Medicine (TOPMED).^28^ Additionally, we screened the Project MinE databrowser to check if the variants of this study have already been described.^29,30^ The maximum frequency is given in Supplemental Table 1 when found in the above databases. We used sorts intolerant from tolerant, Primate AI, MetaLR, MetaSVM and REVEL^31^ scores as prediction tools to assess the biological effect of the mutation. The scores for each variant were extracted from the respective sources when possible. To evaluate the conservation at the specific mutation sites, we used the phyloP100 vertebrate conservation score.^31^

### Selection of investigated genes

The association of the known ‘ALS genes’ with ALS is highly variable ranging from risk, over candidate to Mendelian genes.^32^ The most commonly used classification of ALS genes is based on the Amyotrophic Lateral Sclerosis online Database (ALSoD).^33^ We selected 36 genes from ALSoD which were divided into two groups. Group 1 contains genes of the ALSoD categories ‘definitive ALS gene’, ‘strong evidence’ and ‘moderate evidence’. Group 2 contains genes of the ALSoD category ‘tenuous’. Given the weaker association for the genes in Group 2 with ALS, we report the identified Class 4–Class 5 (C4–C5) variants in this group separately in Supplemental Table 4. For each gene, we tested for the enrichment of pathogenic variants in certain regions to identify mutational hotspots. Accumulation was tested with the *χ*^2^ contingency table test^34^ for genes that had at least one exon with more than 2 mutations. For multiple testing corrections, we used the Benjamini–Hochberg procedure.^35^

### Statistical analysis

Statistical analysis was performed with R 4.2.1.^36^ For the distribution analysis of age at onset and Δ ALSFRS-R/m, we used a two-sided *t*-test. For multiple testing corrections, we used the Benjamini–Hochberg procedure. We used a Cox proportional hazards regression model for the analysis of death from any cause. The model describes the probability of an event or its hazard ratio (HR) for death from any cause for each gene.^37^ We used the Surv() function of the survival package^38^ to create a survival object. We then used the coxph() function from the survival package with standard parameters with sex as a covariate as sex is an independent determinant of survival in ALS.^39-41^ Only genes containing survival data for more than 10 patients were included in the model. *P*-values are from the Wald statistics. The number of events corresponds to reported deaths from any cause within the observation period of 1500 days since disease onset. Global *P*-value corresponds to the overall significance of the model using the likelihood ratio test.^42^

## Results

### Study design and patient cohort

**Figure 1:**
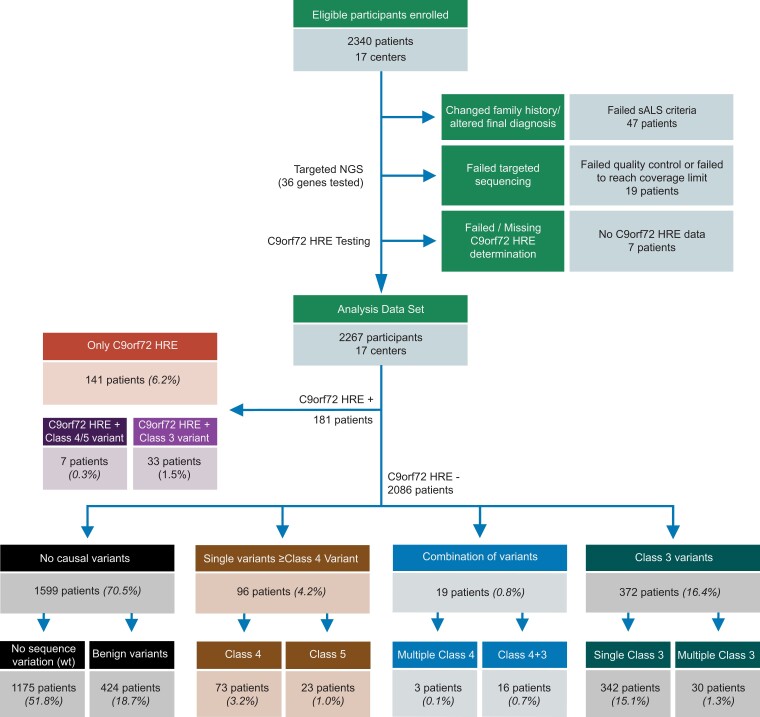
**Study flow chart.** Two thousand three hundred and forty ALS patients of 16 centres of the German MND-NET with a negative family history of ALS were enrolled in the study. Forty-seven patients dropped out due to a change in the family history of ALS or a change in the final diagnosis. Nineteen patients dropped out because quality criteria in sequencing were not met. Seven patients were excluded as no C9orf72 HRE data were available. The final analysis data set contained 2267 patients. All variants were classified according to the ACMG guidelines. C, ACMG variants class; HRE, hexanucleotide repeat expansion; NGS, next-generation sequencing; wt, wild-type.

In this study, we enrolled 2340 sporadic ALS patients from 17 centres of the German MND-NET to screen for variants in 36 ALS-associated genes and the *C9orf72* HRE. Forty-seven patients were excluded because of inconsistent information about diagnosis and/or family history. Nineteen patients were excluded because they did not reach the quality criteria in the next-generation sequencing. For seven patients, no *C9orf72* HRE data were available. Thus, the present analysis included 2267 patients (Fig. 1). The share of females to males was 953:1314 (42:58%) (Supplemental Table 2). The mean age at onset was 60.9 years (±11.1 years), which is very close to the mean age at onset of other European sALS cohorts.^43,44^ Included phenotypes comprised spinal ALS (43.4%), bulbar ALS (18.5%), upper motor neuron predominant ALS (UMN-ALS) (6.9%), lower motor neuron predominant ALS (16.7%), Flail Arm Syndrome (5.1%), Flail Leg Syndrome (1.4%), ALS with FTD (5.6%) and primary lateral sclerosis (2.2%). The mean decrease rate of the ALSFRS-R score^45^ was −0.78 points per month (±0.75) (Supplemental Table 2). Overall, based on the demographic and clinical patient data of our cohort, there was no evidence of a selection bias in this study.

### Investigated genes

An overview of the investigated genes in this study is given in Supplemental Table 1. Despite a large number of ALS-associated genes, a clear convergence of the affected pathways and cellular functions is evident. The genes can be predominantly categorized into four disease-associated mechanisms: (i) protein trafficking, stability and degradation, (ii) RNA processing and nuclear export/import, (iii) cytoskeletal and axonal function and (iv) mitochondrial function, with only minor exceptions (e.g. *VEGFA*).^21,46^ Furthermore, many ALS-associated genes affect cellular function mainly or partially via a loss-of-function mechanism (Supplemental Table 1). For *ARHGEF28*, *FIG4*, *FUS*, *GRN*, *MAPT*, *SPG11* and *TARDBP*, we could demonstrate the enrichment of pathogenic variants as genetic hotspots in specific exons (Supplemental Table 1).

### Cohort analysis

Out of 2267 included patients, we found 181 patients with the pathological *C9orf72* HRE corresponding to roughly 8% of the total cohort, which is higher compared with sALS cohorts of other ethnicities.^6^ From these 181 patients, we identified 7 patients harbouring the *C9orf72* HRE and an additional pathogenic variant (Fig. 1). Thirty-three *C9orf72* HRE patients were identified that had an additional C3 variant. From the *C9orf72* HRE negative patients, 96 patients were identified with a single pathogenic variant (73 patients with a C4 variant and 23 patients with a C5 variant). Nineteen patients showed combinations of variants (3 patients with multiple pathogenic variants, 16 patients with a pathogenic variant and an additional C3 variant). For patients with multiple variants, the clinical characteristics are provided in Supplemental Table 3. Regarding C3 variants, we identified 342 patients with a singular C3 variant and 30 patients with combinations of C3 variants. One thousand one hundred and seventy-five (51.8%) patients showed solely wild-type alleles in all 36 investigated genes (Fig. 1). Four hundred and twenty-four patients showed variants classified as benign or likely benign (C1/C2) or were heterozygous for genes associated with autosomal recessive traits e.g. *ALS2, CFAP410, GLE1, SIGMAR1, SPG11* and *VEGFA*, which were added to the wild-type group, hereafter referred to as reference group (Fig. 1). Two patients were homozygous for mutations in the *GLE1* gene (each one GLE1:c.1422C>A and GLE1:c.5C>G), which were classified as C3 variants, and three patients showed compound heterozygous variants in the *SPG11* gene (each one SPG11:c.2305C>T/SPG11:c.6944A>C, SPG11:c.3320G>C/SPG11:c.6475G>C and SPG11:c.3956T>C/SPG11:c.6907C>G), which were also classified as C3 variants.

### Variant analysis

**Figure 2:**
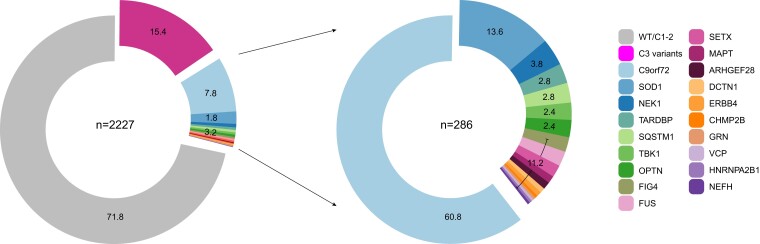
**Pie charts of the abundance of gene variants in all investigated genes.** Patients with multiple Class 4 variants (*n* = 3), multiple Class 3 variants (*n* = 30) and patients harbouring the *C9orf72* HRE and an additional pathogenic variant (*n* = 7) were excluded from the graphical visualization as they could not be assigned to a single gene. The left pie plot presents Class 3/variant of uncertain significance variants as a separate group with 15.4% of all patients falling into this group. The right pie plot shows only Class 4 variants, the most abundant pathogenic variants are in the *C9orf72* gene (*C9orf72* HRE), followed by pathogenic variants in the *SOD1*, *NEK1*, *TARDBP*, *SQSTM1*, *TBK1*, *OPTN*, *FUS*, FIG4, SETX, MAPT, ARHGEF28, DCTN1, ERBB4, CHMP2B, GRN, VCP, HNRNPA2B1 and NEFH genes. *n*, total number of patients included in the respective analysis, numbers in chart slices: percentage of all patients in the respective analysis.

In total, we found 89 pathogenic variants (without the *C9orf72* HRE) of which 31 variants are novel and, to our knowledge, have not been described previously. Four hundred and thirty-seven C3 variants (15.4%) were identified including 103 novel C3 variants (Fig. 2). A few pathogenic variants e.g. SOD1:c.272A>C (p.D91A), as well as some C3 variants that have higher frequencies in the control databases, were recurrent. There were no indications of any family relationships between patients harbouring recurrent variants. The frequency of occurrence of the variants is given in the respective tables (Tables 1 and 3, Supplemental Tables 4 and 5). With C3 variants not considered, we found *C9orf72* HRE (7.81%) to be the most common pathogenic gene variant, followed by pathogenic variants in the *SOD1* (1.75%), *NEK1* (0.49%), *TARDBP* (0.36%), *SQSTM1* (0.36%), *TBK1* (0.31%), *OPTN* (0.31%), *FUS* (0.27%), *FIG4* (0.27%), *SETX* (0.22%), *MAPT* (0.13%), *ARHGEF28* (0.13%), *DCTN1* (0.09%), *ERBB4* (0.09%), *CHMP2B* (0.04%), *GRN* (0.04%), *VCP* (0.04%), *HNRNPA2B1* (0.04%) and *NEFH* (0.04%) genes. In total, 12.74% of all patients showed a *C9orf72* HRE or a pathogenic variant in one of the investigated genes (Fig. 2).

**Table 1:** Overview of pathogenic variants in ALS genes A–N (definitive ALS gene, strong and moderate evidence)

Gene	c.HGVS	p.HGVS	ACMG Class	SNPEff/VEP consensus effect	Transcript	MaxFreq database	No of patients	SIFT	PrimateAI	MetaLR score	MetaSVM score	REVEL score	phyloP100way vertebrate	Known variant	Null variant
ARHGEF28	c.957_963 + 16delinsCG		4	fs&mis&sp_re&int	E8/36	0.000000	1	0	0	0	0	0	0	No	Yes
	c.1180G>T	p.E394*	4	st_gain	E11/36	0.000029	1	0	0	0	0	0	5.8	Yes	Yes
	c.1915A>G	p.T639A	4	mis&sp_re	E16/36	0.000000	1	0.24	0.32	0.01	−0.96	0.05	3.63	No	Yes
	c.1969C>T	p.P657S	4	mis	E16/36	0.000116	1	0.31	0.38	0.51	−0.39	0.22	0.76	Yes	Yes
	c.4903C>T	p.Q1635*	4	st_gain	E35/36	0.000118	1	0	0	0	0	0	2.62	Yes	Yes
CHMP2B	c.27delC	p.T9*fs*5	4	fs	E1/6	0.000000	1	0	0	0	0	0	0	Yes	Yes
ERBB4	c.1718G>A	p.G573D	4	mis&sp_re	E15/28	0.000000	1	0.06	0.87	0.34	−0.49	0.39	7.9	Yes	Yes
	c.3287delG	p.G1096*fs*56	4	fs	E27/28	0.000000	1	0	0	0	0	0	0	No	Yes
FIG4	c.646G>A	p.G216R	4	mis&sp_re	E6/23	0.001094	1	0.69	0.69	0.04	−1.05	0.27	3.79	Yes	Yes
	c.2095C>T	p.R699C	4	mis&sp_re	E18/23	0.000522	2	0	0.62	0.18	−0.77	0.37	1.7	Yes	Yes
	c.2096G>A	p.R699H	4	mis&sp_re	E18/23	0.004739	1	0	0.55	0.17	−0.74	0.34	6.23	Yes	Yes
	c.2467C>T	p.Q823*	4	st_gain	E22/23	0.000375	1	0	0	0	0	0	2.9	Yes	Yes
	c.2695C>T	p.R899*	4	st_gain	E23/23	0.000111	1	0	0	0	0	0	1.27	Yes	Yes
FUS	c.83°C>T	p.S277F	4	mis&sp_re	E8/15	0.000024	1	0.02	0.76	0.16	−0.89	0.14	4.86	Yes	No
	c.1509_1510delAG	p.DR502E*fs*14	4	fs	E14/15	0.000000	1	0	0	0	0	0	0	No	Yes
	c.1529A>G	p.K510R	4	mis	E14/15	0.000000	1	0.03	0.75	0.89	0.87	0.79	2.86	Yes	No
	c.1561C>T	p.R521C	5	mis	E15/15	0.000411	3	0	0.66	0.85	0.37	0.65	2.83	Yes	No
GLE1	c.1706G>A	p.R569H	5	mis	E12/16	0.002358	3	0	0.75	0.78	0.7	0.9	7.98	Yes	No
NEK1	c.304G>T	p.E102*	4	st_gain	E5/36	0.000000	1	0	0	0	0	0	7.58	No	Yes
	c.3107C>G	p.S1036*	4	st_gain	E31/36	0.000366	3	0	0	0	0	0	1.29	Yes	Yes
	c.379C>T	p.R127*	4	st_gain	E6/36	0.000083	2	0	0	0	0	0	3.08	Yes	Yes
	c.546delT	p.N182*fs*27	4	fs	E8/36	0.000000	1	0	0	0	0	0	0	No	Yes
	c.1097_1098delGA	p.R366*fs*6	4	fs	E14/36	0.000000	4	0	0	0	0	0	0	Yes	Yes
	c.1142T>A	p.I381N	4	mis&sp_re	E15/36	0.000012	1	0.02	0.6	0.23	−0.61	0.2	2.92	Yes	Yes
	c.1394G>A	p.W465*	4	st_gain	E17/36	0.000000	1	0	0	0	0	0	1.8	No	No
	c.1911 + 2T>C		4	sp_do&int	I22/35	0.000000	1	0	0	0	0	0	6.74	No	Yes

HGVS, Human Genome Variation Society; c.HGVS, coding DNA reference sequence HGVS notation; p.HGVS, predicted consequences on protein level; ACMG Class, American College of Medical Genetics and Genomics Class; VEP, ensembl variant effect predictor; mis, missense variant; int, intron variant; st_los, stop-loss variant; st_gain, stop-gain variant; fs, frameshift variant; inf_del, in-frame deletion; inf_ins, in-frame insertion; sp_do, splice-donor variant; sp_re, splice region variant; sp_tr, splice tract variant; sp_ac, splice acceptor variant; syn, synonymous variant; 5prUTR, 5 prime UTR variant; cod, coding sequence variant; noncod_ex, non-coding transcript exon variant; nmd, nonsense-mediated mRNA decay variant; E, exon, I, intron; MaxFreq Database, maximum frequency of the variant in one of the databases used; SIFT, sorts intolerant from tolerant, score ≤0.05 is probably deleterious and a score >0.05 is probably tolerated; PrimateAI, threshold of >0.8 is likely pathogenic, <0.6 is likely benign and 0.6–0.8 is intermediate; MetaLR, range between 0 and 1, higher scores are more deleterious; MetaSVM, range between 0 and 1, higher scores are more deleterious; REVEL, range between 0 and 1, higher scores reflect a greater likelihood that a variant is disease-causing; PhyloP100way, conservation score: the greater the score, the more conserved the site, not conserved <1.4, weakly conserved <3.81, conserved >6.8, highly conserved >7.2; Known variant, has already been described in the literature; Null variant, is a null variant (nonsense, frameshift, exon deletion, start loss variant, intronic variant within ±2 bases of the transcript splice site).

**Table 2:** Overview of pathogenic variants in ALS genes O-S (definitive ALS gene, strong and moderate evidence)

Gene	c.HGVS	p.HGVS	ACMG Class	SNPEff/VEP consensus effect	Transcript	MaxFreq Database	No of patients	SIFT	PrimateAI	MetaLR score	MetaSVM score	REVEL score	phyloP100way vertebrate	Known variant	Null variant
OPTN	c.370-1G>A		4	sp_ac&int	I4/14	0.000000	1	0	0	0	0	0	2.76	No	Yes
	c.375delC	p.P125*fs*24	4	fs	E5/15	0.000000	1	0	0	0	0	0	0	Yes	No
	c.381_382_insAG	p.-128*fs*22	5	fs	E5/15	0.007353	1	0	0	0	0	0	0	Yes	Yes
	c.785C>A	p.S262*	4	st_gain	E8/15	0.000065	2	0	0	0	0	0	0.83	Yes	Yes
	c.1583_1584delCT	p.S528*fs*1	4	fs	E14/15	0.000000	2	0	0	0	0	0	0	No	Yes
SOD1	c.115C>G	p.L39V	4	mis	E2/5	0.000009	1	0	0.5	0.99	1.1	0.65	−0.46	Yes	No
	c.131A>G	p.H44R	4	mis	E2/5	0.000009	1	0.21	0.63	0.99	1.06	0.91	8.39	Yes	No
	c.146A>G	p.H49R	4	mis	E2/5	0.000000	1	0	0.69	1	0.89	0.98	8.39	Yes	No
	c.262G>A	p.V88M	4	mis	E4/5	0.000000	1	0	0.64	0.99	1.01	0.86	9.37	Yes	No
	c.272A>C	p.D91A	4	mis	E4/5	0.014384	13	0.04	0.27	0.97	1.2	0.56	0.31	Yes	No
	c.286G>A	p.A96T	4	mis	E4/5	0.000000	1	0.09	0.5	0.99	1.04	0.78	9.37	Yes	No
	c.290A>T	p.D97V	4	mis	E4/5	0.000000	1	0.16	0.21	0.89	0.75	0.53	0.38	Yes	No
	c.313A>T	p.I105F	4	mis	E4/5	0.000000	1	0	0.58	0.98	1.05	0.84	3	Yes	No
	c.341T>C	p.I114T	5	mis	E4/5	0.000105	1	0.04	0.64	0.99	0.97	0.99	7.49	Yes	No
	c.346C>G	p.R116G	5	mis	E4/5	0.000018	8	0	0.63	1	0.89	0.97	5.74	Yes	No
	c.347G>A	p.R116H	4	mis	E4/5	0.000192	1	0.1	0.65	1	0.89	0.95	9.37	Yes	No
	c.352C>G	p.L118V	4	mis	E4/5	0.000000	1	1	0.41	0.94	1.55	0.52	1.75	Yes	No
	c.400G>A	p.E134K	4	mis	E5/5	0.000000	1	0.13	0.66	0.98	1.07	0.83	9.37	Yes	No
	c.400_402delGAA	p.E134del	4	inf_del	E5/5	0.000000	1	0	0	0	0	0	0	No	No
	c.435G>C	p.L145F	5	mis	E5/5	0.000375	5	0.04	0.69	0.99	1.04	0.92	2.58	Yes	No
	c.446T>C	p.V149A	4	mis	E5/5	0.000000	1	0	0.63	0.99	1.02	0.92	7.49	Yes	No
	c.446T>G	p.V149G	4	mis	E5/5	0.000009	1	0	0.52	0.99	1.02	0.95	7.49	Yes	No
SQSTM1	c.754 + 1G>T		4	sp_do&int	I5/7	0.000000	1	0	0	0	0	0	8.86	No	Yes
	c.1175C>T	p.P392L	4	mis	E8/8	0.007903	9	0	0.74	0.63	0.58	0.82	7.87	Yes	No

HGVS, human genome variation society; c.HGVS: coding DNA reference sequence HGVS notation; p.HGVS, predicted consequences on protein level; ACMG Class, American College of Medical Genetics and Genomics Class; VEP, ensembl variant effect predictor; mis, missense variant; int, intron variant; st_los, stop-loss variant; st_gain, stop-gain variant; fs, frameshift variant; inf_del, in-frame deletion; inf_ins, in-frame insertion; sp_do, splice-donor variant; sp_re, splice region variant; sp_tr, splice tract variant; sp_ac, splice acceptor variant; syn, synonymous variant; 5prUTR, 5 prime UTR variant; cod, coding sequence variant; noncod_ex, non-coding transcript exon variant; nmd, nonsense-mediated mRNA decay variant; E, exon; I, intron; MaxFreq Database, maximum frequency of the variant in one of the databases used; SIFT, sorts intolerant from tolerant, score ≤0.05 is probably deleterious and a score >0.05 is probably tolerated; PrimateAI, threshold of >0.8 is likely pathogenic, <0.6 is likely benign, and 0.6–0.8 is intermediate; MetaLR, range between 0 and 1, higher scores are more deleterious; MetaSVM, range between 0 and 1, higher scores are more deleterious; REVEL, range between 0 and 1, higher scores reflect a greater likelihood that a variant is disease-causing; PhyloP100way, conservation score: the greater the score, the more conserved the site, not conserved <1.4, weakly conserved <3.81, conserved >6.8, highly conserved >7.2; Known variant, has already been described in the literature; Null variant, is a null variant (nonsense, frameshift, exon deletion, start loss variant and intronic variant within ±2 bases of the transcript splice site).

**Table 3:** Overview of pathogenic variants in ALS genes T–Z (definitive ALS gene, strong and moderate evidence)

Gene	c.HGVS	p.HGVS	ACMG class	SNPEff/VEP consensus effect	Transcript	MaxFreq database	No of patients	SIFT	PrimateAI	MetaLR score	MetaSVM score	REVEL score	phyloP100way vertebrate	Known variant	Null variant
TARDBP	c.859G>A	p.G287S	4	mis	E6/6	0.000100	1	0.34	0.84	0.49	−0.24	0.42	7.7	Yes	No
	c.881G>T	p.G294V	4	mis	E6/6	0.000029	1	0.1	0.68	0.63	0.12	0.53	4.63	Yes	No
	c.883G>C	p.G295R	4	mis	E6/6	0.000009	1	0.14	0.71	0.64	0.12	0.66	7.13	Yes	No
	c.962C>G	p.A321G	4	mis	E6/6	0.000000	1	0.15	0.71	0.78	0.56	0.56	7.59	Yes	No
	c.1009A>G	p.M337V	5	mis	E6/6	0.000018	1	0.13	0.8	0.81	0.7	0.7	8.95	Yes	No
	c.1144G>A	p.A382T	5	mis	E6/6	0.000066	3	0.13	0.6	0.76	0.3	0.52	2.95	Yes	No
	c.1243_*3delinsATCGATG		4	st_los&fs	E6/6	0.000000	1	0	0	0	0	0	0	No	No
TBK1	c.78_79delAA	p.GR26G*fs*3	4	fs	E2/21	0.000000	1	0	0	0	0	0	0	No	Yes
	c.228 + 1G>A		4	sp_do&int	I3/20	0.000000	1	0	0	0	0	0	8.01	Yes	Yes
	c.1069C>T	p.R357*	4	st_gain	E9/21	0.000023	2	0	0	0	0	0	4.57	Yes	Yes
	c.1341-2delA		4	sp_ac&int	I11/20	0.000000	2	0	0	0	0	0	0	No	Yes
	c.1760 + 1G>C		4	sp_do&int	I16/20	0.000000	1	0	0	0	0	0	8.27	Yes	Yes
	c.1852G>T	p.E618*	4	st_gain	E17/21	0.000000	1	0	0	0	0	0	7.93	No	Yes
	c.1927G>T	p.E643*	4	st_gain	E18/21	0.000000	1	0	0	0	0	0	6.31	Yes	Yes
VCP	c.572G>A	p.R191Q	5	mis	E5/17	0.002100	1	0.02	0.86	0.96	1.07	0.82	7.77	Yes	No

HGVS, human genome variation society; c.HGVS, coding DNA reference sequence HGVS notation; p.HGVS, predicted consequences on protein level; ACMG Class, American College of Medical Genetics and Genomics Class; VEP, ensembl variant effect predictor; mis, missense variant; int, intron variant; st_los: stop-loss variant; st_gain, stop-gain variant; fs, frameshift variant; inf_del, in-frame deletion; inf_ins, in-frame insertion; sp_do, splice-donor variant; sp_re, splice region variant; sp_tr, splice tract variant; sp_ac, splice acceptor variant; syn, synonymous variant; 5prUTR, 5 prime UTR variant; cod, coding sequence variant; noncod_ex, non-coding transcript exon variant; nmd, nonsense-mediated mRNA decay variant; E, exon; I, intron; MaxFreq Database, maximum frequency of the variant in one of the databases used; SIFT, sorts intolerant from tolerant, score ≤0.05 is probably deleterious and a score >0.05 is probably tolerated; PrimateAI, threshold of >0.8 is likely pathogenic, <0.6 is likely benign, and 0.6–0.8 is intermediate; MetaLR, range between 0 and 1, higher scores are more deleterious; MetaSVM, range between 0 and 1, higher scores are more deleterious; REVEL, range between 0 and 1, higher scores reflect a greater likelihood that a variant is disease-causing; PhyloP100way, conservation score: the greater the score, the more conserved the site, not conserved <1.4, weakly conserved <3.81, conserved >6.8 and highly conserved >7.2; Known variant, has already been described in the literature; Null variant, is a null variant (nonsense, frameshift, exon deletion, start loss variant and intronic variant within ±2 bases of the transcript splice site).

### Overview of novel pathogenic variants

**Figure 3:**
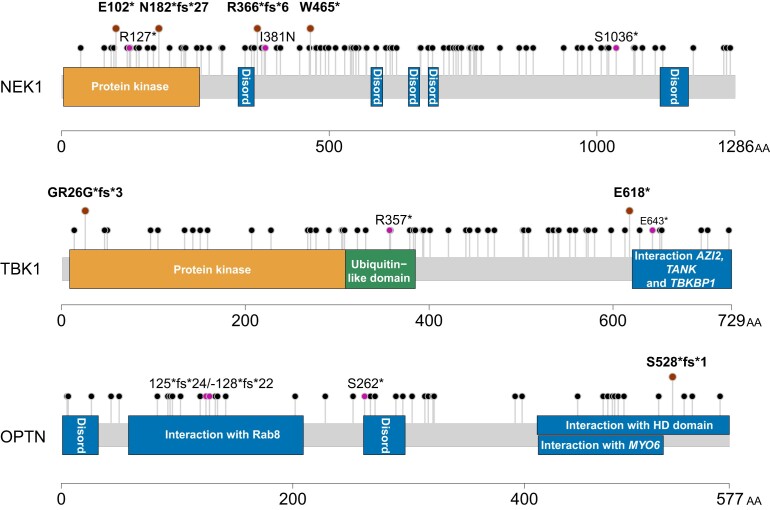
**Graphical visualization of pathogenic variants in selected genes of our cohort.** Variants are depicted in the lollipop plot overlying the respective gene structure. Lollipops with brown filling represent novel pathogenic variants, lollipops with purple filling represent known pathogenic variants found in our cohort and lollipops with black filling represent known variants in the literature. The horizontal bars represent functional protein domains. Arabic numerals correspond to amino acid numbers. Disord, disordered protein region according to MobiDB-lite rules; fs, frameshift; AA, amino acid; AZI2, 5-azacytidine induced 2; TANK, TRAF family member-associated NF-kappa-B activator; TBKBP1, TBK1 binding protein 1; MYO6, myosin VI; HD domain, histidine-aspartate domain.

We found novel pathogenic variants in the following 16 genes: *NEK1, SPG11, MAPT, TBK1, ARHGEF28, OPTN, SETX, DCTN1, ERBB4, FUS, GRN, HNRNPA2B1, SOD1, SQSTM1, TARDBP* and *ALS2*. We identified 16 frameshift, 9 splice-site, 4 stop-variants and 2 in-frame deletions. For the three genes with the highest number of novel pathogenic variants *NEK1*, *TBK1* and *OPTN,* we show a graphical visualization of the pathogenic variants in their respective gene products (Fig. 3). We classified novel and known variants according to the ACMG guidelines and provide the maximum frequencies in the reference databases, as well as various prediction and conservation scores for all, found C4–C5 variants in Tables 1–3. For the genes with the ALSoD category ‘tenuous’, we provide an overview of the detected C4–C5 variants in Supplemental Table 4. All C3 variants found in this cohort are provided in Supplemental Table 5.

### Clinical features and demographic data of different subgroups

**Figure 4:**
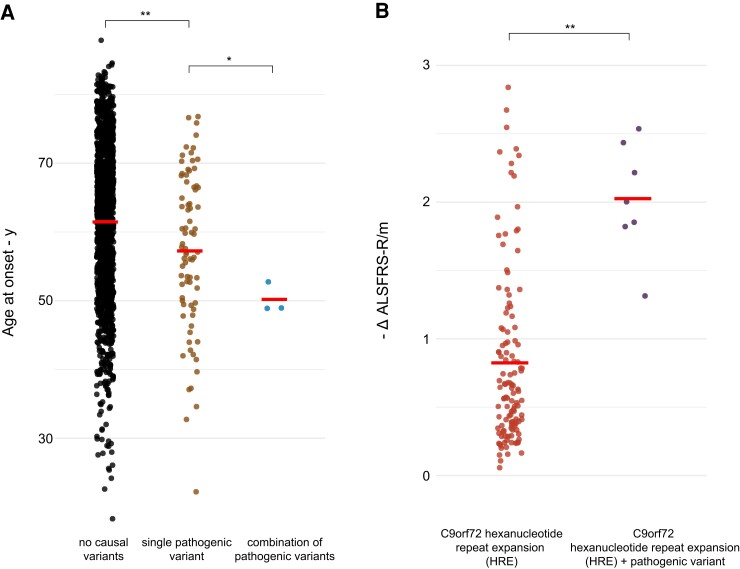
**Point diagram of the distribution of demographic and clinical patient data in different groups.** (**A**) Distribution of age at onset shows a significantly younger age at onset for patients with a single pathogenic variant [without the *C9orf72* hexanucleotide repeat expansion (HRE), *n* = 96] compared with the reference group (*n* = 1599) and younger age at onset for patients with combinations of pathogenic variants (*n* = 3) compared with patients with a single pathogenic variant. (**B**) A higher Δ ALSFRS-R/m for patients harbouring the *C9orf72* HRE and an additional pathogenic variant (*n* = 7) than for patients with the *C9orf72* HRE alone (*n* = 141) are shown (two-sided *t*-test followed by Benjamini–Hochberg corrections *P < 0.05, *P < 0.005).

Analysing clinical features and demographic data of different groups [no causal variants, single pathogenic variants (without the *C9orf72* HRE), combination of pathogenic variants in different genes], we could show a significantly younger age at onset (*P*-adj < 0.004) for patients with a single pathogenic variant in comparison to patients without a causal variant. Despite a small number of patients, we could confirm a significantly younger age at onset (*P*-adj < 0.009) for patients with combinations of pathogenic variants in different genes than for patients with a single pathogenic variant which has previously been described^47^ (Fig. 4A). The Δ ALSFRS-R/m between the three groups was not significantly different (data not shown). For patients harbouring the *C9orf72* HRE, we could not detect an earlier age at onset for patients with an additional pathogenic variant than for patients with the *C9orf72* HRE alone. However, patients with the *C9orf72* HRE and an additional pathogenic variant showed a significantly higher decrease rate of ALSFRS-R/m (*P*-adj < 0.003) in comparison to patients with the *C9orf72* HRE alone (Fig. 4B). A higher decrease rate of the ALSFRS-R/m score is associated with a shorter overall survival.^48^

### Gene-specific HRs for death from any cause show an increased HR for the *C9orf72* HRE and a decreased HR for pathogenic *SOD1* variants

**Figure 5:**
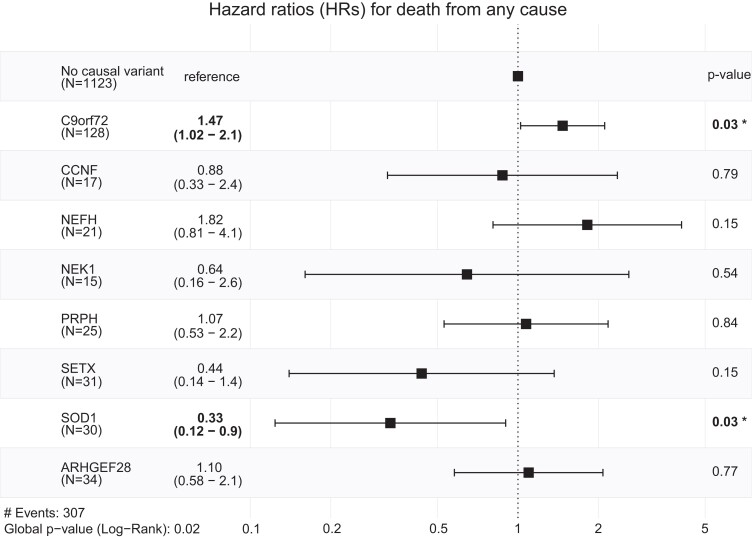
**Forrest diagram of gene-wise HRs for death from any cause.** Significant HRs and their corresponding *P*-values are visualized in bold. In brackets, the 95% confidence interval is given. Patients without a causal variant serve as references (*P*-value: *P*-values from the Wald statistics * *P* < 0.05). *N*, number of individuals with survival data harbouring pathogenic variants in the respective gene; # events, total number of reported deaths from any cause within the observation period (global *P*-value: overall significance of the model using the likelihood ratio test).

Survival data were available for 1424 patients, comprising an observation period of 1500 days after symptom onset. All patients with C3 variants or combinations of pathogenic variants were excluded from this analysis. We found that most genes harbour very diverging variants concerning their HRs for death from any cause, giving less meaningful and vague overall HRs per gene. However, for genes harbouring more homozygous variants and for the *C9orf72* HRE we found an increased HR of 1.47 (95% confidence interval 1.02–2.1) for the *C9orf72* HRE and a decreased HR of 0.33 (95% confidence interval 0.12–0.9) for pathogenic variants in the *SOD1* gene in comparison to patients without a causal gene variant (Fig. 5).

## Discussion

In this study, we genetically and clinically characterized a large sALS cohort from Central Europe, to provide a comprehensive summary of the frequencies of known pathogenic gene variants in the context of sALS in Central Europe and to identify novel variants. Thirty-one novel C4/C5 variants (without the *C9orf72* HRE) were found which could be helpful in mechanistic research.

For the *C9orf72* gene, we found a higher prevalence of the *C9orf72* HRE in Caucasian sALS patients (∼8%) compared with other populations: Japan (0.4%),^49^ China (0.9%),^50^ Latin America (3.4%) and North America (5.2%).^51^ A similar frequency for the *C9orf72* HRE (∼7%) has previously been described in a Caucasian cohort.^52^ This underlines the importance of *C9orf72* HRE testing even in the absence of a positive familial history for ALS/FTD. While 96 patients were identified with a single C4–C5 variant (without the *C9orf72* HRE) (4.2%), 342 patients were detected with a C3 variant which corresponds to around 15% of the cohort. The high number of C3 variants poses a particular challenge to the clinician, due to their common occurrence and their unknown impact on the disease. However, with expanding genetic and clinical data in the field of MNDs, more C3 variants will be classified into clinically more meaningful classes like benign and pathogenic variants. While the majority of cases showed single pathogenic variants in one of the ALS genes, 10 patients had combinations of pathogenic variants in more than one tested gene. The prognostic assessment of such combinations is difficult due to their innumerable possible combinations. However, we could show that the age at onset for patients with pathogenic variants in multiple genes is significantly younger than for patients with a singular C4/C5 variant. Additionally, we could show that the Δ ALSFRS-R/m is higher for patients harbouring the *C9orf72* HRE and an additional pathogenic variant than for patients with the *C9orf72* HRE alone. These findings are in line with a previous study suggesting that multiple mutations may have synergistic clinical effects^53^ and corroborate the theory of oligogenic causation in a proportion of ALS cases. The co-occurrence of pathogenic variants is also important for the design of gene-specific therapeutic trials, where stratification of these patient groups might be necessary. It also suggests that combinatorial therapeutic approaches targeting different disease mechanisms may be a promising strategy. Finally, we analysed survival data showing that for most genes the HR for death from any cause is very heterogeneous. Here, a gene-wise ALS-related prognosis estimation is of limited information, and a variant-wise approach for HRs should be preferred whenever possible. However, given the limited survival data, considering each gene variant individually, a gene-wise HR estimation for genes with clinically more homogenous phenotypes like *C9orf72* HRE can give valuable information. Thus, we could show a higher HR for the *C9orf72* HRE and a lower HR for pathogenic variants in the *SOD1* gene.

Altogether we identified 227 patients (∼10% of our cohort) that would be eligible for one of the three gene-specific therapies (*SOD1/FUS/C9orf72*), who would have remained undetected if the patient selection for genetic screening was solely based on the presence of a positive family history for ALS. So, our findings corroborate that genetic testing should be made available to all sALS patients after respective counselling. With the increase of genetic and clinical data for sALS, preferably in the form of a central database, a better prognosis estimation based on variant-wise approaches would be possible and more of the numerous C3 variants could be translated into benign or pathogenic variants.

## Supplementary Material

fcad152_Supplementary_Data

## Data Availability

The data of this study are available from the corresponding author, upon reasonable request.

## Abbreviations

ACMG =: American College of Medical Genetics and Genomics
ALS =: amyotrophic lateral sclerosis
ALSFRS-R =: amyotrophic lateral sclerosis functional rating scale-revised
ALSoD =: Amyotrophic Lateral Aclerosis online Database
C =: class
cod =: coding sequence variant
E =: exon
ExAC =: Exome Aggregation Consortium
fs =: frameshift variant
FTD =: frontotemporal dementia
FUS =: FUsed in Sarcoma
GnomAD =: genome aggregation database
HGVS =: Human Genome Variation Society
HR =: hazard ratio
HRE =: hexanucleotide repeat expansion
I =: intron
IN =: inherited neuropathies
inf_del =: in-frame deletion
inf_ins =: in-frame insertion
int =: intron variant
LoF =: loss of function
mis =: missense variant
MND =: motoneuron disease
MND-NET =: German Network for Motor Neuron Disease
nmd =: nonsense-mediated mRNA decay variant
noncod_ex =: non-coding transcript exon variant
PLS =: primary lateral sclerosis
sALS =: sporadic amyotrophic lateral sclerosis
SIFT =: sorts intolerant from tolerant
SNV =: single-nucleotide variant
sp_ac =: splice acceptor variant
sp_do =: splice-donor variant
sPLS =: sporadic primary lateral sclerosis
sp_re =: splice region variant
sp_tr =: splice tract variant
SOD1 =: superoxide dismutase 1
st_gain =: stop-gain variant
st_los =: stop-loss variant
syn =: synonymous variant
TOPMED =: trans-omics for precision medicine
UMN =: upper motor neuron
UMN-ALS =: upper motor neuron amyotrophic lateral sclerosis
VEP =: ensembl variant effect predictor
5prUTR =: 5 prime UTR variant

## References

[1] Brown RH , Al-Chalabi A. Amyotrophic lateral sclerosis. N Engl J Med. 2017;377(2):162–172. 28700839 10.1056/NEJMra1603471

[2] Korobeynikov VA , Lyashchenko AK, Blanco-Redondo B, Jafar-Nejad P, Shneider NA. Antisense oligonucleotide silencing of FUS expression as a therapeutic approach in amyotrophic lateral sclerosis. Nat Med. 2022;28(1):104–116. 35075293 10.1038/s41591-021-01615-z PMC8799464

[3] Miller TM , Cudkowicz ME, Genge A, et al Trial of antisense oligonucleotide tofersen for *SOD1* ALS. N Engl J Med. 2022;387(12):1099–1110. 36129998 10.1056/NEJMoa2204705

[4] Müller K , Brenner D, Weydt P, et al Comprehensive analysis of the mutation spectrum in 301 German ALS families. J Neurol Neurosurg Psychiatry. 2018;89(8):817–827. 29650794 10.1136/jnnp-2017-317611

[5] Liu ZJ , Lin HX, Wei Q, et al Genetic spectrum and variability in Chinese patients with amyotrophic lateral sclerosis. Aging Dis. 2019;10(6):1199–1206. 31788332 10.14336/AD.2019.0215 PMC6844596

[6] Zou Z-Y , Zhou Z-R, Che C-H, Liu C-Y, He R-L, Huang H-P. Genetic epidemiology of amyotrophic lateral sclerosis: A systematic review and meta-analysis. J Neurol Neurosurg Psychiatry. 2017;88(7):540–549. 28057713 10.1136/jnnp-2016-315018

[7] Ludolph A , Drory V, Hardiman O, et al A revision of the El Escorial criteria-2015. Amyotroph Lateral Scler Frontotemporal Degener. 2015;16(5–6):291–292. 26121170 10.3109/21678421.2015.1049183

[8] Pringle C , Hudson A, Munoz D, Kiernan J, Brown W, Ebers G. Primary lateral sclerosis: Clinical features, neuropathology and diagnostic criteria. Brain. 1992;115(2):495–520. 1606479 10.1093/brain/115.2.495

[9] Zondler L , Müller K, Khalaji S, et al Peripheral monocytes are functionally altered and invade the CNS in ALS patients. Acta Neuropathol. 2016;132(3):391–411. 26910103 10.1007/s00401-016-1548-y

[10] DeJesus-Hernandez M , Mackenzie IR, Boeve BF, et al Expanded GGGGCC hexanucleotide repeat in noncoding region of C9ORF72 causes chromosome 9p-linked FTD and ALS. Neuron. 2011;72(2):245–256. 21944778 10.1016/j.neuron.2011.09.011 PMC3202986

[11] Renton AE , Majounie E, Waite A, et al A hexanucleotide repeat expansion in C9ORF72 is the cause of chromosome 9p21-linked ALS-FTD. Neuron. 2011;72(2):257–268. 21944779 10.1016/j.neuron.2011.09.010 PMC3200438

[12] Southern EM . Detection of specific sequences among DNA fragments separated by gel electrophoresis. J Mol Biol. 1975;98(3):503–517. 1195397 10.1016/s0022-2836(75)80083-0

[13] Cunningham F , Allen JE, Allen J, et al Ensembl 2022. Nucleic Acids Res. 2022;50(D1):D988–D995. 34791404 10.1093/nar/gkab1049 PMC8728283

[14] Cingolani P , Platts A, Wang LL, et al A program for annotating and predicting the effects of single nucleotide polymorphisms, SnpEff: SNPs in the genome of *Drosophila melanogaster* strain w1118; iso-2; iso-3. Fly (Austin). 2012;6(2):80–92. 22728672 10.4161/fly.19695 PMC3679285

[15] Richards S , Aziz N, Bale S, et al Standards and guidelines for the interpretation of sequence variants: A joint consensus recommendation of the American College of Medical Genetics and Genomics and the Association for Molecular Pathology. Genet Med. 2015;17(5):405–423. 25741868 10.1038/gim.2015.30 PMC4544753

[16] Solomon BD , Nguyen A-D, Bear KA, Wolfsberg TG. Clinical genomic database. Proc Natl Acad Sci USA. 2013;110(24):9851–9855. 23696674 10.1073/pnas.1302575110 PMC3683745

[17] Amberger JS , Bocchini CA, Schiettecatte F, Scott AF, Hamosh A. OMIM.org: Online Mendelian Inheritance in Man (OMIM®), an online catalog of human genes and genetic disorders. Nucleic Acids Res. 2015;43(D1):D789–D798. 25428349 10.1093/nar/gku1205 PMC4383985

[18] Kopanos C , Tsiolkas V, Kouris A, et al Varsome: The human genomic variant search engine. Bioinformatics. 2019;35(11):1978–1980. 30376034 10.1093/bioinformatics/bty897 PMC6546127

[19] Todd TW , Petrucelli L. Modelling amyotrophic lateral sclerosis in rodents. Nat Rev Neurosci. 2022;23(4):231–251. 35260846 10.1038/s41583-022-00564-x

[20] Kim G , Gautier O, Tassoni-Tsuchida E, Ma XR, Gitler AD. ALS genetics: Gains, losses, and implications for future therapies. Neuron. 2020;108(5):822–842. 32931756 10.1016/j.neuron.2020.08.022 PMC7736125

[21] Weishaupt JH , Hyman T, Dikic I. Common molecular pathways in amyotrophic lateral sclerosis and frontotemporal dementia. Trends Mol Med. 2016;22(9):769–783. 27498188 10.1016/j.molmed.2016.07.005

[22] Karczewski KJ , Francioli LC, Tiao G, et al The mutational constraint spectrum quantified from variation in 141,456 humans. Nature. 2020;581(7809):434–443. 32461654 10.1038/s41586-020-2308-7 PMC7334197

[23] Karczewski KJ , Weisburd B, Thomas B, et al The ExAC browser: Displaying reference data information from over 60 000 exomes. Nucleic Acids Res. 2017;45(D1):D840–D845. 27899611 10.1093/nar/gkw971 PMC5210650

[24] Phan L , Jin Y, Zhang H, et al ALFA: Allele frequency aggregator. National Center for Biotechnology Information, US National Library of Medicine. 2020:10.

[25] 1000 Genomes Project Consortium . A map of human genome variation from population scale sequencing. Nature. 2010;467(7319):1061–1073. 20981092 10.1038/nature09534 PMC3042601

[26] UK10K Consortium . The UK10K project identifies rare variants in health and disease. Nature. 2015;526(7571):82–90. 26367797 10.1038/nature14962 PMC4773891

[27] Moayyeri A , Hammond CJ, Hart DJ, Spector TD. The UK Adult Twin Registry (TwinsUK Resource). Twin Res Hum Genet. 2013;16(1):144–149. 23088889 10.1017/thg.2012.89 PMC3927054

[28] Seplyarskiy VB , Soldatov RA, Koch E, et al Population sequencing data reveal a compendium of mutational processes in the human germ line. Science. 2021;373(6558):1030–1035. 34385354 10.1126/science.aba7408 PMC9217108

[29] Van Rheenen W , Pulit SL, Dekker AM, et al Project MinE: Study design and pilot analyses of a large-scale whole-genome sequencing study in amyotrophic lateral sclerosis. Eur J Hum Genet. 2018;26(10):1537–1546. 29955173 10.1038/s41431-018-0177-4 PMC6138692

[30] van der Spek RAA , van Rheenen W, Pulit SL, Kenna KP, van den Berg LH, Veldink JH. The project MinE databrowser: Bringing large-scale whole-genome sequencing in ALS to researchers and the public. Amyotroph Lateral Scler Frontotemporal Degener. 2019;20(5–6):432–440. 31280677 10.1080/21678421.2019.1606244 PMC7893599

[31] Liu X , Li C, Mou C, Dong Y, Tu Y. dbNSFP v4: A comprehensive database of transcript-specific functional predictions and annotations for human nonsynonymous and splice-site SNVs. Genome Med. 2020;12(1):103. 33261662 10.1186/s13073-020-00803-9 PMC7709417

[32] Corcia P , Couratier P, Blasco H, et al Genetics of amyotrophic lateral sclerosis. Rev Neurol (Paris). 2017;173(5):254–262. 28449881 10.1016/j.neurol.2017.03.030

[33] Lill CM , Abel O, Bertram L, Al-Chalabi A. Keeping up with genetic discoveries in amyotrophic lateral sclerosis: The ALSoD and ALSGene databases. Amyotroph Lateral Scler. 2011;12(4):238–249. 21702733 10.3109/17482968.2011.584629

[34] Hope AC . A simplified Monte Carlo significance test procedure. J R Stat Soc Ser B (Methodol). 1968;30(3):582–598.

[35] Benjamini Y , Hochberg Y. Controlling the false discovery rate: A practical and powerful approach to multiple testing. J R Stat Soc Ser B (Methodol). 1995;57(1):289–300.

[36] R Core Team . R: A language and environment for statistical computing. Vienna, Austria: R Foundation for Statistical Computing; 2013.

[37] Andersen PK , Gill RD. Cox's regression model for counting processes: A large sample study. Ann Stat. 1982;10:1100–1120.

[38] Borgan O , Langholz B, Samuelsen SO, Goldstein L, Pogoda J. Exposure stratified case-cohort designs. Lifetime Data Anal. 2000;6(1):39–58. 10763560 10.1023/a:1009661900674

[39] Tang L , Ma Y, Liu X-L, Chen L, Fan D-S. Better survival in female *SOD1*-mutant patients with ALS: A study of *SOD1*-related natural history. Transl Neurodegener. 2019;8(1):1–10. 30637102 10.1186/s40035-018-0142-8 PMC6325854

[40] Tysnes O-B , Vollset SE, Larsen JP, Aarli JA. Prognostic factors and survival in amyotrophic lateral sclerosis. Neuroepidemiology. 1994;13(5):226–235. 7969707 10.1159/000110384

[41] Trojsi F , Siciliano M, Femiano C, et al Comparative analysis of C9orf72 and sporadic disease in a large multicenter ALS population: The effect of male sex on survival of C9orf72 positive patients. Front Neurosci. 2019;13:485. 31156370 10.3389/fnins.2019.00485 PMC6534038

[42] Solomon DL . A note on the non-equivalence of the Neyman-Pearson and generalized likelihood ratio tests for testing a simple null versus a simple alternative hypothesis. Am Stat. 1975;29(2):101–102.

[43] Mehta PR , Jones AR, Opie-Martin S, et al Younger age of onset in familial amyotrophic lateral sclerosis is a result of pathogenic gene variants, rather than ascertainment bias. J Neurol Neurosurg Psychiatry. 2019;90(3):268–271. 30270202 10.1136/jnnp-2018-319089 PMC6518463

[44] Dorst J , Schuster J, Dreyhaupt J, et al Effect of high-caloric nutrition on serum neurofilament light chain levels in amyotrophic lateral sclerosis. J Neurol Neurosurg Psychiatry. 2020;91(9):1007–1009. 32788256 10.1136/jnnp-2020-323372

[45] Cedarbaum JM , Stambler N, Malta E, et al The ALSFRS-R: A revised ALS functional rating scale that incorporates assessments of respiratory function. BDNF ALS Study Group (Phase III). J Neurol Sci. 1999;169(1–2):13–21. 10540002 10.1016/s0022-510x(99)00210-5

[46] Hardiman O , Al-Chalabi A, Chio A, et al Amyotrophic lateral sclerosis. Nat Rev Dis Primers. 2017;3(1):1–19. 10.1038/nrdp.2017.85 29052611

[47] Shepheard SR , Parker MD, Cooper-Knock J, et al Value of systematic genetic screening of patients with amyotrophic lateral sclerosis. J Neurol Neurosurg Psychiatry. 2021;92(5):510–518. 33589474 10.1136/jnnp-2020-325014 PMC8053339

[48] Dorst J , Chen L, Rosenbohm A, et al Prognostic factors in ALS: A comparison between Germany and China. J Neurol. 2019;266(6):1516–1525. 30923935 10.1007/s00415-019-09290-4

[49] Ogaki K , Li Y, Atsuta N, et al Analysis of C9orf72 repeat expansion in 563 Japanese patients with amyotrophic lateral sclerosis. Neurobiol Aging. 2012;33(10):2527.e11–2527.e16. 10.1016/j.neurobiolaging.2012.05.011 22727276

[50] Chen Y , Lin Z, Chen X, et al Large C9orf72 repeat expansions are seen in Chinese patients with sporadic amyotrophic lateral sclerosis. Neurobiol Aging. 2016;38:217.e15–217.e22. 10.1016/j.neurobiolaging.2015.11.016 26725464

[51] Brown CA , Lally C, Kupelian V, Flanders WD. Estimated prevalence and incidence of amyotrophic lateral sclerosis and SOD1 and C9orf72 genetic variants. Neuroepidemiology. 2021;55(5):342–353. 34247168 10.1159/000516752

[52] Majounie E , Renton AE, Mok K, et al Frequency of the C9orf72 hexanucleotide repeat expansion in patients with amyotrophic lateral sclerosis and frontotemporal dementia: A cross-sectional study. Lancet Neurol. 2012;11(4):323–330. 22406228 10.1016/S1474-4422(12)70043-1 PMC3322422

[53] Yilmaz R , Weishaupt K, Valkadinov I, Knehr A, Brenner D, Weishaupt JH. Quadruple genetic variants in a sporadic ALS patient. Mol Genet Genomic Med. 2022;10(7):e1953. 35426263 10.1002/mgg3.1953 PMC9266611

