# Supplementary material for: Spectrum and frequency of genetic variants in sporadic amyotrophic lateral sclerosis
Source: Brain Commun. 2023 May 9;5(3):fcad152. doi: 10.1093/braincomms/fcad152 (PMC10202555; doi:10.1093/braincomms/fcad152)
Supplement: fcad152_Supplementary_Data [file fcad152_supplementary_data.pdf]

Supplemental Table 1 Overview of the investigated ALS-associated genes in this study. ALS: Amyotrophic lateral sclerosis, PLS: Primary lateral sclerosis, SCA: Spinocerebellar ataxia, FTD: Frontotemporal dementia, CMT: Charcot–Marie–Tooth, IN: Inherited Neuropathies, IBM: Inclusion body myositis, CM: Congenital myopathy. #: number, MOI: mode of inheritance, LoF: loss of function, Unk: unknown, E: exon, ALFA: Allele Frequency Aggregator, ESP6500: Exome Sequencing Project, oth: Other (population not assigned), afr: African/African American, eas: East Asian, sas: South Asian, asj: Ashkenazi Jewish, fin: Finnish, amr: Latino/Admixed American, (chi-squared contingency table test followed by Benjamini-Hochberg corrections * p<0.05, **p<0.005, *** p<0.0005).

Supplemental Table 2 Characteristics of participants. In total 2267 participants were analyzed. The female to male ratio was 42:58%, the mean age at onset was 60.9y (±11.1y), and the percentages of the 8 different phenotypes: spinal ALS, bulbar ALS, UMN-predominant ALS, LMN-predominant ALS, Flail Arm Syndrome, Flail Leg Syndrome, ALS-FTD, PLS were 43.4%, 18.5%, 6.9%,16.7%, 5.1%, 1.4%, 5.6%, and 2.2%. Δ ALSFRS-R/m was calculated as follows: Δ ALSFRS-R/m = (48-ALSFRS-R score at visit) / (date of the visit- date of onset) (months), * Data are mean ±SD, ‖ Data were available for 1886 participants, ¶ Data were available for 1785 participants, ALS: Amyotrophic lateral sclerosis, UMN: upper motor neuron, LMN: lower motor neuron, ALS-FTD: Amyotrophic lateral sclerosis with frontotemporal dementia, PLS: Primary lateral sclerosis.

Supplemental Table 3 Characteristics of participants with multiple variants. (**A**) Characteristics of participants with multiple pathogenic variants. (**B**) Characteristics of participants with a pathogenic variant and an additional Class 3 variant. f: female, m: male, HGVS: Human Genome Variation Society, c.HGVS: coding DNA reference sequence HGVS notation, p.HGVS: predicted consequences on protein level, ACMG Class: American College of Medical Genetics and Genomics Class, Δ ALSFRS-R/m = (48-ALSFRS-R score at visit) / (date of the visit- date of onset) (months).

Supplemental Table 4 Overview of pathogenic variants in investigated ALS genes (ALS gene association: tenuous evidence). 14 genes fall into this category. HGVS: Human Genome Variation Society, c.HGVS: coding DNA reference sequence HGVS notation, p.HGVS: predicted consequences on protein level, g.HGVS: genomic reference sequence HGVS notation, ACMG Class: American College of Medical Genetics and Genomics Class, VEP: Ensembl Variant Effect Predictor, mis: missense variant, int: intron variant, st_los: stop-loss variant, st_gain: stop-gain variant, fs: frameshift variant, inf_del: in-frame deletion, inf_ins: in-frame insertion, sp_do: splice-donor variant, sp_re: splice region variant, sp_tr: splice tract variant, sp_ac: splice acceptor variant, syn: synonymous variant, 5prUTR: 5 prime UTR variant, cod: coding sequence variant, noncod_ex: non-coding transcript exon variant, nmd: nonsense-mediated mRNA decay variant. E: exon, I: intron, MaxFreq Database: Maximum frequency of the variant in one of the databases used, SIFT: sorts intolerant from tolerant, score <=0.05 is probably deleterious and a score>0.05 is probably tolerated. PrimateAI: score a threshold of > 0.8 is likely pathogenic, < 0.6 is likely benign, and 0.6–0.8 is intermediate, MetaLR: range between 0 to 1, higher scores are more deleterious, MetaSVM: range between 0 to 1, higher scores are more deleterious, REVEL: range between 0 to 1, higher scores reflect a greater likelihood that a variant is disease-causing, Polyphen2 HDIV/HVAR: Score >=0.957 is probably damaging, 0.453-0.956 is possibly damaging, <=0.452 is probably benign, PhyloP100way: conservation score: the greater the score, the more conserved the site, not conserved <1.4, weakly conserved <3.81, conserved >6.8, highly

conserved >7.2, Known variant: has already been described in the literature, Null variant: is a null variant (nonsense, frameshift, exon deletion, start loss variant, intronic variant within ±2 bases of the transcript splice site).

Supplemental Table 5 Overview of variants of uncertain significance. (**A**) ALS genes with ALS gene association: definitive ALS gene, strong, and moderate evidence). (**B**) ALS genes with ALS gene association: tenuous evidence. HGVS: Human Genome Variation Society, c.HGVS: coding DNA reference sequence HGVS notation, p.HGVS: predicted consequences on protein level, g.HGVS: genomic reference sequence HGVS notation, ACMG Class: American College of Medical Genetics and Genomics Class, VEP: Ensembl Variant Effect Predictor, mis: missense variant, int: intron variant, st_los: stop-loss variant, st_gain: stop-gain variant, fs: frameshift variant, inf_del: in-frame deletion, inf_ins: in-frame insertion, sp_do: splice-donor variant, sp_re: splice region variant, sp_tr: splice tract variant, sp_ac: splice acceptor variant, syn: synonymous variant, 5prUTR: 5 prime UTR variant, cod: coding sequence variant, noncod_ex: non-coding transcript exon variant, nmd: nonsense-mediated mRNA decay variant. E: exon, I: intron, MaxFreq Database: Maximum frequency of the variant in one of the databases used, SIFT: sorts intolerant from tolerant, score <=0.05 is probably deleterious and a score>0.05 is probably tolerated. PrimateAI: score a threshold of > 0.8 is likely pathogenic, < 0.6 is likely benign, and 0.6–0.8 is intermediate, MetaLR: range between 0 to 1, higher scores are more deleterious, MetaSVM: range between 0 to 1, higher scores are more deleterious, REVEL: range between 0 to 1, higher scores reflect a greater likelihood that a variant is disease-causing, Polyphen2 HDIV/HVAR: Score >=0.957 is probably damaging, 0.453-0.956 is possibly damaging, <=0.452 is probably benign, PhyloP100way: conservation score: the greater the score, the more conserved the site, not conserved <1.4, weakly conserved <3.81, conserved >6.8, highly conserved >7.2, Known variant: has already been described in the literature, Null variant: is a null variant (nonsense, frameshift, exon deletion, start loss variant, intronic variant within ±2 bases of the transcript splice site). ‖ variants found homozygous in a gene associated with an autosomal recessive trait. *, ¶, ‡ variants found compound heterozygous in genes associated with autosomal recessive traits.

Supplemental Table 1 ALS genes investigated in this study, ordered alphabetically

Gene	Localisation	ENSEMBL Transcript	Clinical Traits	MOI	LoF	ALSod Category	Gene classified in Group 1: definitive ALS gene, strong and moderate evidence	Putative gene function	# Variants of uncertain significance (% all variants)	# Pathogenic variants (% all variants)	Most abundant pathogenic variant (Highest frequency in reference databases)	BS1 cut-off frequency	Hotspot Exon pathogenic variants p
ALS2	2q33.1	ENST00000264276	ALS,PLS	AR	1	Tenuous	2	Protein trafficking, stability, degradation	29 (60.4%)	2 (4.2%)	ALS2:c.4831C>T (0.0001, NCBI ALFA)	0.0001	-
ANG	14q11.2	ENST00000397990	ALS	AD	0	Moderate	1	RNA processing and nuclear export	1 (12.5%)	0 (0%)	-	0.0028	-
ARHGEF28	5q13.2	ENST00000513042	ALS	AD,AR	1	Moderate	1	RNA processing and nuclear export	23 (27.7%)	5 (6.0%)	ARHGEF28:c.4903C>T (0.0001, ESP6500 European American)	0.0001	E16/36 **
C9orf72	9p21.2	ENST00000380003	ALS,FTD	AD	1	HRE Definitive	1	Protein trafficking, stability, degradation	-	-	-	0.0001	-
CCNF	16p13.3	ENST00000397066	ALS,FTD	AD	1	Strong	1	Protein trafficking, stability, degradation	12 (29.2%)	0 (0%)	-	0.0002	-
CFAP410	21q22.3	ENST00000339818	ALS	AR	1	Strong	1	Cytoskeletal and axonal function	0 (0%)	0 (0%)	-	0.0006	-
CHCHD10	22q11.23	ENST00000484558	ALS,FTD,CM	AD	0	Definitive	1	Mitochondria	3 (50%)	0 (0%)	-	0.0002	-
CHMP2B	3p11.2	ENST00000263780	ALS, FTD	AD	1	Moderate	1	Protein trafficking, stability, degradation	2 (14.3%)	1 (7.1%)	CHMP2B:c.27delC (0)	0.0001	-
DCTN1	2p13.1	ENST00000628224	ALS, IN	AD,AR	1	Tenuous	2	Cytoskeletal and axonal function	28 (63.6%)	2 (4.5%)	DCTN1:c.2731dupG (0)	0.0067	-
ERBB4	2q34	ENST00000342788	ALS	AD	1	Moderate	1	Other	17 (62.9%)	2 (7.4%)	ERBB4:c.3287delG (0)	0.0008	-
FIG4	6q21	ENST00000230124	ALS,CMT	AD	1	Moderate	1	Protein trafficking, stability, degradation	24 (66.7%)	6 (16.7%)	FIG4:c.2096G>A (0.0047, gnomAD v2 Genoms controls (oth))	0.0089	E18/23 *
FUS	16p11.2	ENST00000254108	ALS	AD,AR	1	Definitive	1	RNA processing and nuclear export/import	17 (58.6%)	6 (20.7%)	FUS:c.1561C>T (0.0004, gnomAD v2 Genoms controls (afr))	0.0007	E14/15 **
GLE1	9q34.11	ENST00000309971	ALS	AR	1	Moderate	1	RNA processing and nuclear export	26 (83.9%)	3 (9.7%)	GLE1:c.1706G>A (0.0024, gnomAD v2 Genoms controls (oth))	0.012	-
GRN	17q21.31	ENST00000053867	FTD	AD,AR	1	Tenuous	2	Protein trafficking, stability, degradation	19 (76%)	2 (8%)	GRN:c.1603C>T (0.0001, gnomAD v2 Exoms controls (eas))	0.0004	E12/13 **
HNRNPA1	12q13.13	ENST00000340913	ALS,FTD, IBM	AD	Unk	Definitive	1	RNA processing and nuclear export	4 (57.1%)	0 (0%)	-	0.0001	-
HNRNPA2B1	7p15.2	ENST00000679123	ALS,FTD, IBM	AD	1	Tenuous	2	RNA processing and nuclear export	6 (50%)	1 (8.3%)	HNRNPA2B1:c.8_10delAAA (0)	0.0001	-
MAPT	17q21.31	ENST00000415613	FTD	AD,AR	1	Tenuous	2	Cytoskeletal and axonal function	16 (35.6%)	4 (8.9%)	MAPT:c.428dupC (0)	0.0007	E5/14 *
MATR3	5q31.2	ENST00000394805	ALS, CM	AD	1	Tenuous	2	RNA processing and nuclear export	27 (79.4%)	0 (0%)	-	0.0002	-
NEFH	22q12.2	ENST00000310624	ALS, CMT	AD	Unk	Tenuous	2	Cytoskeletal and axonal function	24 (43.6%)	1 (1.82%)	NEFH:c.883G>A (0.0001, gnomAD v2 Exoms controls (sas))	0.0001	-
NEK1	4q33	ENST00000507142	ALS	AD	1	Definitive	1	Cytoskeletal and axonal function	77 (70.6%)	14 (12.8%)	NEK1:c.3107C>G (0.0004, ESP6500 European American)	0.0039	-
OPTN	10p13	ENST00000378747	ALS,FTD	AD,AR	1	Definitive	1	Protein trafficking, stability, degradation	11 (40.7%)	7 (25.9%)	OPTN:c.381_382_insAG (0.0074, gnomAD v3 Genoms controls (asj))	0.0037	-
PFN1	17p13.2	ENST00000225655	ALS	AD	0	Definitive	1	Cytoskeletal and axonal function	5 (100%)	0 (0%)	-	0.0001	-
PRPH	12q13.12	ENST00000257860	ALS	AD,AR	Unk	Tenuous	2	Cytoskeletal and axonal function	3 (6.9%)	0 (0%)	-	0.0001	-
SETX	9q34.13	ENST00000224140	ALS, SCA	AD,AR	1	Tenuous	2	RNA processing and nuclear export	57 (50%)	5 (4.4%)	SETX:c.6085A>G (0.0013, gnomAD v3 Genoms controls (oth))	0.0036	-
SIGMAR1	9p13.3	ENST00000277010	ALS,FTD	AR	1	Tenuous	2	Protein trafficking, stability, degradation	1 (11.1%)	0 (0%)	-	0.0002	-
SOD1	21q22.11	ENST00000270142	ALS	AD,AR	1	Definitive	1	Protein trafficking, stability, degradation	1 (2.2%)	43 (95.5%)	SOD1:c.272A>C (0.0144, gnomAD v2 Genoms (fin))	0.012	-
SPG11	15q21.1	ENST00000261866	ALS, CMT	AR	1	Tenuous	2	Cytoskeletal and axonal function	67 (38.3%)	7 (4%)	SPG11:c.5255delT (0.0002, gnomAD v3 Genoms controls (amr))	0.0003	E40/40 **
SQSTM1	5q35.3	ENST00000389805	ALS,FTD	AD	1	Moderate	1	Protein trafficking, stability, degradation	11 (23.9%)	10 (21.7%)	SQSTM1:c.1175C>T (0.0079, gnomAD v3 Genoms controls (amr))	0.0015	-
TAF15	17q12	ENST00000605844	ALS	AD	1	Tenuous	2	RNA processing and nuclear export	21 (45.6%)	0 (0%)	-	0.0001	-
TARDBP	1p36.22	ENST00000240185	ALS	AD	1	Definitive	1	RNA processing and nuclear export	2 (15.3%)	9 (69.2%)	TARDBP:c.859G>A (0.0001, NCBI ALFA)	0.0002	E6/6 ***
TBK1	12q14.2	ENST00000331710	ALS,FTD	AD	1	Definitive	1	Protein trafficking, stability, degradation	25 (59.5%)	9 (21.4%)	TBK1:c.1069C>T (0)	0.0003	-
TUBA4A	2q35	ENST00000248437	ALS,FTD	AD	1	Strong	1	Cytoskeletal and axonal function	3 (100%)	0 (0%)	-	0.0001	-
UBQLN2	Xp11.21	ENST00000338222	ALS,FTD	XLD	1	Definitive	1	Protein trafficking, stability, degradation	7 (100%)	0 (0%)	-	0.0065	-
VAPB	20q13.32	ENST00000475243	ALS	AD	1	Definitive	1	Protein trafficking, stability, degradation	3 (13.64%)	0 (0%)	-	0.0001	-
VCP	9p13.3	ENST00000358901	ALS,FTD, IBM	AD	1	Definitive	1	Protein trafficking, stability, degradation	1 (33.3%)	1 (33.3%)	VCP:c.572G>A (0.0021, NCBI ALFA)	0.0001	-
VEGFA	6p21.1	ENST00000372055	ALS	AR	1	Tenuous	2	Other	8 (66.7%)	0 (0%)	-	0.0004	-
VPS54	2p15p14	ENST00000272322	ALS	AD	1	Tenuous	2	Protein trafficking, stability, degradation	14 (36.8%)	0 (0%)	-	0.0001	-

**Supplemental Table 2 Characteristics of the participants**	
**Characteristic**	**Total (N=2267)**
Female sex (%)	953 (42%)
Age at onset - y * ‖	60.9 (+/-11.1)
Phenotype of motoneuron disease - no. (%)	
Spinal ALS	985/2267 (43.4%)
Bulbar ALS	419/2267 (18.5%)
UMN-predominant ALS	157/2267 (6.9%)
LMN-predominant ALS	379/2267 (16.7%)
Flail Arm Syndrome	116/2267 (5.1%)
Flail Leg Syndrome	32/2267 (1.4%)
ALS-FTD	128/2267 (5.6%)
PLS	51/2267 (2.2%)
Δ ALSFRS-R/m * ¶	-0.78 (+/- 0.75)

Supplemental Table 3 - A Characteristics of participants with multiple pathogenic variants

Age at onset - y	sex	C9orf72 hexanucleotide repeat expansion (HRE)	Variant 1 Gene	Variant 1 c.HGVS	Variant 1 p.HGVS	Variant 1 ACMG Class	Variant 2 Gene	Variant 2 c.HGVS	Variant 2 ACMG Class	Variant 2 ACMG Class	Phenotype of motoneuron disease	Δ ALSFRS-R/m
52.7	m	no	NEK1	c.1142T>A	p.I381N	4	ARHGEF28	c.1915A>G	p.T639A	4	Spinal ALS	0.47
48.9	m	no	TARDBP	c.859G>A	p.G287S	4	ARHGEF28	c.1969C>T	p.P657S	4	Spinal ALS	1.59
48.9	m	no	NEK1	c.3446G>T	p.R366*fs*6	4	SETX	c.479_498+9del	-	4	Flail Arm Syndrome	0.59
43	m	yes	SOD1	c.272A>C	p.D91A	4				5	Spinal ALS	2.44
51.4	m	yes	TBK1	c.1341-2delA	-	4				5	ALS-FTD	2.22
68.9	f	yes	SQSTM1	c.1175C>T	p.P392L	4				5	Bulbar ALS	2.00
54.8	f	yes	TBK1	c.1069C>T	p.R357*	4				5	Spinal ALS	1.85
63.3	f	yes	SOD1	c.1175C>T	p.R116G	5				5	Spinal ALS	1.82
49.7	f	yes	GRN	c.1414G>C	p.A472P	4				5	ALS-FTD	2.54
53.8	m	yes	MAPT	c.1293delinsTT	p.C432Lfs*9	4				5	Bulbar ALS	1.31

Supplemental Table 3 - B Characteristics of participants with a pathogenic variant and an additional C3 variant

Age at onset - y	sex	C9orf72 hexanucleotide repeat expansion (HRE)	Variant 1 Gene	Variant 1 c.HGVS	Variant 1 p.HGVS	Variant 1 ACMG Class	Variant 2 Gene	Variant 2 c.HGVS	Variant 2 ACMG Class	Variant 2 ACMG Class	Phenotype of motoneuron disease	Δ ALSFRS-R/m
62.5	m	no	TBK1	c.228+1G>A		4	NEK1	c.3637C>A	p.H1213N	3	ALS-FTD	0.19
52.3	f	no	SOD1	c.435G>C	p.L145F	4	NEK1	c.89A>G	p.Y30C	3	Spinal ALS	NA
59.8	f	no	NEFH	c.883G>A	p.V295M	4	ARHGEF28	c.4645G>A	p.E1549K	3	PLS	0.18
55.8	m	no	NEK1	c.3107C>G	p.S1036*	4	FIG4	c.2097-10C>G	-	3	Flail Arm Syndrome	0.27
46.8	f	no	SOD1	c.313A>T	p.I105F	4	DCTN1	c.3134T>C	p.I1045T	3	Spinal ALS	0.35
65.6	f	no	SQSTM1	c.1175C>T	p.P392L	4	PRPH	c.1376G>A	p.R459H	3	Spinal ALS	NA
57.6		no	SETX	c.8C>T	p.T3I	4	FUS	c.170_172delCTT	p.SY57_SY58delinsY	3	Spinal ALS	NA
62	m	no	FIG4	c.2095	p.R699C	4	DCTN1	c.673C>T	p.I1045T	3	LMN-predominant ALS	1.48
53.6	f	no	ARHGEF28	c.1180G>T	p.E394*	4	MAPT	c.683C>G	p.S228C	3	Bulbar ALS	1.78
40.3	m	no	SOD1	c.272A>C	p.D91A	4	FIG4	c.2116G>A	p.V706I	3	Spinal ALS	0.94
48.6		no	NEK1	c.1097_1098delGA	p.R366*fs*6	4	TBK1	c.1760+4_1760+7delAGTA	-	3	Spinal ALS	0.65
60.2	f	no	TARDBP	c.881G>T	p.G294V	4	MATR3	c.2533A>G	p.K845E	3	Bulbar ALS	0.74
38.3	m	no	SQSTM1	c.754+1G>T	-	4	SETX	c.5322G>T	p.Q1774H	3	Bulbar ALS	1.09
55	m	no	TARDBP	c.1243_*3delinsATCGATG	-	4	DCTN1	c.59C>T	p.A20V	3	ALS-FTD	0.63
63.4	f	no	SOD1	c.262G>A	p.V88M	4	ERBB4	c.1879G>A	p.G627S	3	LMN-predominant ALS	NA
52.4	m	no	HNRNPA2B1	c.8_10delAAA	p.KT3_KT4delinsT	4	NEFH	c.1510_1530del	p.PPA504_EAA510del	3	Spinal ALS	0.22

Supplemental Table 4 Overview of pathogenic variants in ALS genes (tenuous evidence)

Gene	c.HGVS	p.HGVS	g.HGVS	ACMG Class (C)	SNPEff / VEP consensus effect	Transcript	MaxFreq Database	Frequency (no of patients)	SIFT	PrimateAI	MetaLR score	MetaSVM score	REVEL score	Polyphen2 HDIV score	Polyphen2 HVAR score	phyloP100 way vertebrate	Known Variant	Null variant
ALS2	c.4271delA	p.Q1424*fs*3	2:g.201709890del	4	fs	E27/34	0.000000	1	0	0.00	0.00	0.00	0.00	0.00	0.00	0.00	no	yes
	c.4831C>T	p.R1611W	2:g.201704461G>A	4	mis	E32/34	0.000100	1	0	0.94	0.28	-0.50	0.80	1.00	1.00	7.95	yes	no
DCTN1	c.150_176del	p.TLF50_WVG59delinsT	2:g.74378103_74378129del	4	inf_del	E2/32	0.000000	1	0	0.00	0.00	0.00	0.00	0.00	0.00	0.00	no	no
	c.2731dupG	p.E911G*fs*3	2:g.74366277dup	4	fs	E23/32	0.000000	1	0	0.00	0.00	0.00	0.00	0.00	0.00	0.00	yes	no
GRN	c.1414G>C	p.A472P	17:g.44352341G>C	4	mis&sp_re	E12/13	0.000000	1	0	0.74	0.83	0.91	0.93	1.00	1.00	7.87	no	yes
	c.1603C>T	p.R535*	17:g.44352530C>T	4	st_gain	E12/13	0.000111	1	0	0.00	0.00	0.00	0.00	0.00	0.00	0.63	yes	yes
HNRNPA2B1	c.8_10delAAA	p.KT3_KT4delinsT	7:g.26197864_26197866del	4	sp_re&inf_del&nmd	E2/13	0.000000	1	0	0.00	0.00	0.00	0.00	0.00	0.00	0.00	no	yes
MAPT	c.428dupC	p.P143P*fs*19	17:g.45983232dup	4	fs	E5/14	0.000000	1	0	0.00	0.00	0.00	0.00	0.00	0.00	0.00	no	yes
	c.895dupC	p.L299P*fs*52	17:g.45983699dup	4	fs	E5/14	0.000000	1	0	0.00	0.00	0.00	0.00	0.00	0.00	0.00	no	yes
	c.1293delinsTT	p.C432Lfs*9	17:g.45989988delinsTT	4	fs&syn	E7/14	0.000000	1	0	0.00	0.00	0.00	0.00	0.00	0.00	0.00	no	yes
	c.1828-1G>A	-	17:g.46010309G>A	4	sp_ac&int	I10/13	0.000000	1	0	0.00	0.00	0.00	0.00	0.00	0.00	9.35	no	yes
NEFH	c.883G>A	p.V295M	22:g.29481145G>A	4	mis&sp_re	E1/4	0.000091	1	0.03	0.81	0.73	0.58	0.54	0.00	0.00	3.66	yes	yes
SETX	c.6421_6422delCA	p.Q2141*fs*23	9:g.132283391_132283392del	4	fs	E19/26	0.000000	1	0	0.00	0.00	0.00	0.00	0.00	0.00	0.00	no	yes
	c.479_498+9del	-	9:g.132342681_132342709del	4	fs&sp_do&cod&int	E5/26	0.000000	1	0	0.00	0.00	0.00	0.00	0.00	0.00	0.00	no	yes
	c.7641dupT	p.-2548*fs*1	9:g.132264633dup	4	fs	E26/26	0.000023	1	0	0.00	0.00	0.00	0.00	0.00	0.00	0.00	yes	yes
	c.6085A>G	p.K2029E	9:g.132295893T>C	4	mis	E15/26	0.001269	1	0.01	0.57	0.49	-0.12	0.58	0.00	0.00	2.98	yes	no
	c.8C>T	p.T3I	9:g.132349421G>A	4	mis	E3/26	0.000000	1	0.03	0.55	0.63	0.46	0.60	1.00	0.91	4.09	yes	no
SPG11	c.5255delT	p.F1752*fs*86	15:g.44584429del	5	fs	E30/40	0.000213	1	0	0.00	0.00	0.00	0.00	0.00	0.00	0.00	yes	yes
	c.7152-1_7156delGATATA	-	15:g.44563299_44563304del	4	fs&int&sp_ac&cod	E40/40	0.000000	1	0	0.00	0.00	0.00	0.00	0.00	0.00	0.00	no	yes
	c.3040C>T	p.L1014F	15:g.44613535G>A	4	mis&sp_re	E17/40	0.000000	1	0.01	0.48	0.69	0.40	0.57	0.99	0.80	2.75	no	yes
	c.2652G>A	p.W884*	15:g.44620372C>T	4	st_gain	E15/40	0.000000	1	0	0.00	0.00	0.00	0.00	0.00	0.00	6.20	no	yes
	c.7255_7256dupAA	p.K2419K*fs*7	15:g.44563197_44563198dup	4	fs	E40/40	0.000000	1	0	0.00	0.00	0.00	0.00	0.00	0.00	0.00	no	yes
	c.2208delC	p.N736*fs*3	15:g.44626367del	4	fs	E11/40	0.000000	1	0	0.00	0.00	0.00	0.00	0.00	0.00	0.00	no	yes
	c.1621C>T	p.Q541*	15:g.44633619G>A	4	st_gain	E8/40	0.000065	1	0	0.00	0.00	0.00	0.00	0.00	0.00	7.33	yes	yes

Supplemental Table 5 - A Overview of variants of uncertain significance in ALS genes (definitive ALS gene, strong and moderate evidence)

Gene	c.HGVS	p.HGVS	g.HGVS	ACMG Class (C)	SNPEff / VEP consensus effect	Transcript	MaxFreq Database	Frequency (no of patients)	SIFT	PrimateAI	MetaLR score	MetaSVM score	REVEL score	Polyphen2 HDIV score	Polyphen2 HVAR score	phyloP100way vertebrate	Known Variant	Null variant
ANG	c.3G>A	p.M1I	14:g.20693567G>A	3	start_lost	E2/2	0.003448	1	0.01	0	0.59	0.1	0.65	0.89	0.44	4.54	yes	yes
ARHGEF28	c.145C>T	p.R49C	5:g.73749948C>T	3	mis	E3/36	0.000841	1	0	0.48	0.1	-1.09	0.19	1	1	2.63	yes	no
	c.232G>A	p.G78S	5:g.73752959G>A	3	mis	E4/36	0.000033	1	0.17	0.6	0.08	-1.06	0.16	1	0.95	4.84	yes	no
	c.805C>T	p.R269W	5:g.73776661C>T	3	mis	E6/36	0.003448	1	0	0.67	0.1	-0.98	0.28	1	1	2.1	yes	no
	c.1453G>A	p.D485N	5:g.73846293G>A	3	mis	E12/36	0.000431	1	0.08	0.44	0.03	-1.1	0.03	0.43	0.04	2.99	yes	no
	c.1663C>T	p.R555C	5:g.73849003C>T	3	mis	E13/36	0.003165	3	0	0.58	0.18	-0.85	0.28	1	1	6.35	yes	no
	c.1679_1680delinsTG	p.S560L	5:g.73849019_73849020inv	3	mis	E13/36	0.000000	1	0	0	0	0	0	0	0	0	no	no
	c.1709G>T	p.R570L	5:g.73849049G>T	3	mis	E13/36	0.000012	1	0.29	0.35	0.07	-0.99	0.13	0.92	0.37	0.93	yes	no
	c.1804_1806del	p.E602del	5:g.73857669_73857671del	3	inf_del	E15/36	0.000000	1	0	0	0	0	0	0	0	0	no	no
	c.1886G>A	p.R629K	5:g.73857751G>A	3	mis	E15/36	0.000834	1	0	0.74	0.06	-1.1	0.19	0.57	0.55	9.56	yes	no
	c.2281_2283delinsAGT	p.G761S	5:g.73868004_73868006delinsAGT	3	mis	E19/36	0.000000	1	1	0	0	0	0	0	0	0	no	no
	c.2425+3G>A	-	5:g.73868230G>A	3	sp_re&int	I20/35	0.000044	1	0	0	0	0	0	0	0	0	yes	no
	c.2686T>C	p.C896R	5:g.73873118T>C	3	mis	E22/36	0.000015	1	0	0.78	0.42	-0.15	0.39	0.73	0.44	4.13	no	no
	c.3020C>T	p.P1007L	5:g.73883849C>T	3	mis	E24/36	0.000000	1	0	0.84	0.64	0.42	0.82	1	1	7.52	no	no
	c.3089C>G	p.A1030G	5:g.73885883C>G	3	mis	E25/36	0.004065	1	0	0.55	0.66	0.33	0.73	0.99	0.84	7.52	yes	no
	c.3089C>T	p.A1030V	5:g.73885883C>T	3	mis	E25/36	0.001092	1	0	0.58	0.67	0.4	0.81	1	0.96	7.52	yes	no
	c.3200C>T	p.T1067M	5:g.73885994C>T	3	mis	E25/36	0.000831	1	0	0.53	0.33	-0.69	0.21	0.97	0.54	5.82	yes	no
	c.3361G>A	p.D1121N	5:g.73887653G>A	3	mis	E26/36	0.000000	1	0	0.8	0.53	0.1	0.4	0.61	0.8	9.48	yes	no
	c.4307A>G	p.E1436G	5:g.73909557A>G	3	mis	E34/36	0.005063	2	0	0.37	0.24	-0.81	0.21	1	0.94	4.63	yes	no
	c.4645G>A	p.E1549K	5:g.73909895G>A	3	mis&sp_re	E34/36	0.000213	1	0.19	0.42	0.14	-0.77	0.12	0.99	0.77	3.25	yes	no
	c.4648-8T>G	-	5:g.73911267T>G	3	sp_re&int	I34/35	0.000000	1	0	0	0	0	0	0	0	0	no	no
CCNF	c.205C>T	p.H69Y	16:g.2432994C>T	3	mis	E3/17	0.000027	1	0.11	0.74	0.04	-1.12	0.14	0.82	0.24	4.34	yes	no
	c.208G>A	p.A70T	16:g.2432997G>A	3	mis	E3/17	0.000065	1	0.37	0.48	0.03	-1.08	0.03	0.08	0.05	1.37	yes	no
	c.248C>T	p.P83L	16:g.2433037C>T	3	mis	E3/17	0.000111	1	0.02	0.6	0.15	-0.91	0.5	1	0.98	7.41	yes	no
	c.307G>A	p.A103T	16:g.2435834G>A	3	mis	E4/17	0.000008	1	0.15	0.83	0.06	-1.12	0.06	0.57	0.17	6.19	yes	no
	c.728G>C	p.S243T	16:g.2439777G>C	3	mis	E8/17	0.000009	1	0.05	0.36	0.05	-1.08	0.11	0.61	0.08	3.24	yes	no
	c.889_891del	p.Q297del	16:g.2443760_2443762del	3	inf_del	E9/17	0.000000	1	0	0	0	0	0	0	0	0	yes	no
	c.1043C>T	p.P348L	16:g.2445571C>T	3	mis	E10/17	0.000066	1	0	0.58	0.09	-1.12	0.16	1	0.91	4.64	yes	no
	c.1322A>C	p.H441P	16:g.2449385A>C	3	mis	E12/17	0.000025	1	0.02	0.51	0.05	-1.01	0.15	0.91	0.56	5.28	yes	no
	c.1677C>G	p.H559Q	16:g.2453499C>G	3	mis	E15/17	0.000000	1	0.46	0.46	0.04	-1.08	0.03	0.01	0	-0.93	yes	no
	c.1715+9C>G	-	16:g.2453546C>G	3	int	I15/16	0.000167	1	0	0	0	0	0	0	0	0	yes	no
	c.1727A>G	p.N576S	16:g.2455406A>G	3	mis	E16/17	0.000100	1	0.16	0.49	0.06	-1.08	0.06	0.09	0.02	6.91	yes	no
	c.2326G>A	p.E776K	16:g.2456985G>A	3	mis	E17/17	0.000015	1	0.03	0.36	0.04	-1	0.04	0	0	3.13	yes	no
CHCHD10	c.12_26del	p.GSR4_SAA9delinsG	22:g.23767849_23767863del	3	inf_del	E1/4	0.000000	1	0	0	0	0	0	0	0	0	no	no
	c.221G>A	p.S74N	22:g.23767414C>T	3	mis	E2/4	0.000065	1	0	0.7	0.26	-0.44	0.2	0	0	5.88	yes	no
	c.408T>C	p.H136=	22:g.23766129A>G	3	sp_re&syn	E3/4	0.000065	1	0	0	0	0	0	0	0	0	yes	no
CHMP2B	c.38T>A	p.V13E	3:g.87240702T>A	3	mis	E2/6	0.000000	1	0.13	0.82	0.46	-0.31	0.38	0.03	0.04	7.32	no	no
	c.425-4C>T	-	3:g.87253400C>T	3	sp_re&int	I4/5	0.000919	1	0	0	0	0	0	0	0	0	yes	no
ERBB4	c.35G>A	p.S12N	2:g.212538496C>T	3	mis	E1/28	0.000478	1	0.57	0.72	0.32	-0.57	0.27	0.53	0.58	4.4	yes	no
	c.268G>T	p.A90S	2:g.211947583C>A	3	mis	E3/28	0.000519	1	0	0.89	0.33	-0.59	0.73	0	0	7.91	yes	no
	c.562C>T	p.R188C	2:g.211750699G>A	3	mis	E5/28	0.000064	1	0.01	0.51	0.61	0.29	0.64	0	0	2.71	yes	no
	c.847T>C	p.Y283H	2:g.211722429A>G	3	mis	E7/28	0.000000	1	0	0.96	0.22	-0.63	0.61	0	0	9.25	no	no
	c.1708C>T	p.H570Y	2:g.211673172G>A	3	mis	E14/28	0.000015	1	1	0.63	0.03	-0.99	0.07	0	0	3.26	yes	no
	c.1720C>A	p.P574T	2:g.211665474G>T	3	mis	E15/28	0.000480	1	0	0.76	0.22	-0.63	0.29	0	0	6.8	yes	no
	c.1879G>A	p.G627S	2:g.211657821C>T	3	mis	E16/28	0.000375	1	0.05	0.6	0.66	0.41	0	0	0	4.03	yes	no
	c.2087A>C	p.E696A	2:g.211624037T>G	3	mis	E18/28	0.000181	1	0	0.86	0.38	-0.37	0.74	0	0	8.02	yes	no
	c.2495T>G	p.M832R	2:g.211431093A>C	3	mis	E21/28	0.000000	1	0.34	0.91	0.22	-0.4	0.6	0	0	6.22	yes	no
	c.2639G>A	p.G880E	2:g.211430949C>T	3	mis	E21/28	0.000100	1	0.02	0.93	0.29	-0.44	0.63	0	0	7.88	yes	no
	c.3153C>A	p.S1051R	2:g.211387975G>T	3	mis	E26/28	0.000000	1	0.06	0.68	0.4	-0.13	0.35	0.99	0.96	6.89	yes	no
	c.3182G>A	p.G1061E	2:g.211387946C>T	3	mis&sp_re	E26/28	0.000349	1	0.42	0.8	0.38	-0.41	0.31	1	1	3.83	yes	yes
	c.3189G>C	p.Q1063H	2:g.211387145C>G	3	mis	E27/28	0.000015	1	0.13	0.67	0.43	-0.41	0.39	0	0	1.45	yes	no
	c.3238C>A	p.P1080T	2:g.211387096G>T	3	mis	E27/28	0.000100	1	0.13	0.5	0.29	-0.62	0.28	0	0	6.19	yes	no
	c.3341C>A	p.P1114Q	2:g.211386993G>T	3	mis	E27/28	0.000000	1	0.8	0.35	0.12	-0.97	0.15	0	0	4.27	no	no
	c.3446G>T	p.G1149V	2:g.211386888C>A	3	mis	E27/28	0.002358	1	0.03	0.77	0.62	0.27	0.63	0	0	7.2	yes	no
	c.3494C>G	p.P1165R	2:g.211384048G>C	3	mis	E28/28	0.000023	1	0.07	0.82	0.52	-0.02	0.44	0	0	9.52	yes	no
FIG4	c.101C>T	p.T34M	6:g.109715112C>T	3	mis	E2/23	0.000524	2	0	0.68	0.49	0.09	0.39	1	1	5.82	yes	no
	c.122T>C	p.I41T	6:g.109715133T>C	3	mis	E2/23	0.004065	10	0	0.82	0.67	0.51	0.84	1	1	5.73	yes	no
	c.772T>G	p.S258A	6:g.109738450T>G	3	mis	E7/23	0.000000	1	0.08	0.68	0.33	-0.52	0.41	1	0.96	5.9	no	no
	c.1137+5G>A	-	6:g.109743777G>A	3	sp_re&int	I10/22	0.000000	1	0	0	0	0	0	0	0	0	no	no
	c.1389-5C>T	-	6:g.109763932C>T	3	sp_re&int	I12/22	0.000059	1	0	0	0	0	0	0	0	0	yes	no

	c.1601A>G	p.Y534C	6:g.109766746A>G	3	mis	E15/23	0.000213	1	0	0.87	0.4	-0.02	0.77	1	1	8.76	yes	no
	c.1880C>A	p.T627K	6:g.109777051C>A	3	mis	E16/23	0.000375	1	0.92	0.36	0.02	-0.98	0.08	0	0	2.34	yes	no
	c.2069T>C	p.F690S	6:g.109786422T>C	3	mis	E18/23	0.000066	1	0.01	0.72	0.16	-0.82	0.51	1	0.93	7.41	yes	no
	c.2097-10C>G	-	6:g.109789584C>G	3	sp_tr&int	I18/22	0.004065	2	0	0	0	0	0	0	0	0	yes	no
	c.2116G>A	p.V706I	6:g.109789613G>A	3	mis	E19/23	0.000140	1	0.16	0.35	0.03	-1.07	0.08	0	0	0.87	yes	no
	c.2200G>A	p.E734K	6:g.109791395G>A	3	mis	E20/23	0.000233	1	0.15	0.67	0.12	-1.04	0.25	0.56	0.17	7.78	yes	no
	c.2256G>C	p.E752D	6:g.109791451G>C	3	mis	E20/23	0.000116	1	0.29	0.68	0.1	-1.03	0.26	0.72	0.22	1.47	yes	no
	c.2573A>G	p.D858G	6:g.109825114A>G	3	mis	E23/23	0.000000	1	0.02	0.58	0.2	-0.65	0.36	1	0.79	5.5	no	no
FUS	c.14-9C>G	-	16:g.31182389C>G	3	sp_tr&int	I1/14	0.000164	1	0	0	0	0	0	0	0	0	yes	no
	c.47C>T	p.A16V	16:g.31182521C>T	3	mis	E3/15	0.001821	1	0.12	0.6	0.63	0.22	0.45	0.7	0.32	4	yes	no
	c.170_172del	p.SY57_SY58delinsY	16:g.31182644_31182646del	3	inf_del	E3/15	0.000000	1	0	0	0	0	0	0	0	0	yes	no
	c.188A>G	p.N63S	16:g.31182662A>G	3	mis&sp_re	E3/15	0.006329	2	0.81	0.57	0.12	-0.96	0.2	0	0	0.72	yes	no
	c.197A>G	p.Y66C	16:g.31183864A>G	3	mis	E4/15	0.000000	1	0	0.74	0.73	0.58	0.69	1	1	7.81	no	no
	c.317C>T	p.P106L	16:g.31183984C>T	3	mis	E4/15	0.000116	3	0.07	0.5	0.29	-0.61	0.32	0.32	0.1	5.43	yes	no
	c.630G>C	p.Q210H	16:g.31185045G>C	3	mis	E6/15	0.000270	2	0.09	0.65	0.86	0.72	0.54	1	0.99	0.4	yes	no
	c.742C>T	p.R248C	16:g.31185157C>T	3	mis	E6/15	0.000060	1	0.12	0.74	0.91	0.87	0.6	0.01	0	3.83	yes	no
	c.1142A>G	p.N381S	16:g.31190115A>G	3	mis	E11/15	0.000000	1	0.55	0.53	0.04	-1.05	0.02	0.07	0.02	3.4	yes	no
	c.1201_1203del	p.G401del	16:g.31190307_31190309del	3	inf_del	E12/15	0.000000	1	0	0	0	0	0	0	0	0	no	no
	c.1292C>T	p.P431L	16:g.31190398C>T	3	mis&sp_re	E12/15	0.004065	2	0.01	0.7	0.29	-0.52	0.52	1	1	7.54	yes	yes
	c.1415G>A	p.R472H	16:g.31190984G>A	3	mis	E14/15	0.000000	1	0.2	0.76	0.76	0.39	0.42	0.02	0.01	4.93	no	no
GLE1I	c.5C>G	p.P2R	9:g.128504810C>G	3	mis	E1/16	0.026316	6	0	0.72	0.28	-0.57	0.27	0.07	0.03	3.71	yes	no
	c.116G>A	p.C39Y	9:g.128508892G>A	3	mis	E2/16	0.001079	2	0.06	0.65	0.18	-0.92	0.21	0.06	0.01	5.63	yes	no
	c.322-9C>A	-	9:g.128515520C>A	3	sp_tr&int	I2/15	0.000009	1	0	0	0	0	0	0	0	0	yes	no
	c.416A>C	p.H139P	9:g.128515623A>C	3	mis	E3/16	0.000000	1	0.01	0.54	0.27	-0.63	0.38	0.94	0.46	5.52	no	no
	c.562C>G	p.R188G	9:g.128522797C>G	3	mis	E4/16	0.000066	1	0	0.23	0.31	-0.7	0.23	0.97	0.51	2.08	yes	no
	c.679C>T	p.R227C	9:g.128523628C>T	3	mis	E6/16	0.001567	4	0	0.6	0.44	-0.15	0.28	1	0.92	4.51	yes	no
	c.877A>T	p.I293F	9:g.128523826A>T	3	mis	E6/16	0.000047	1	0.02	0.55	0.39	-0.28	0.16	0.91	0.24	4.3	yes	no
	c.946C>T	p.R316W	9:g.128525240C>T	3	mis	E7/16	0.000389	1	0	0.35	0.18	-0.74	0.2	0.72	0.03	4.14	yes	no
	c.947G>A	p.R316Q	9:g.128525241G>A	3	mis	E7/16	0.001888	1	1	0.36	0.08	-0.96	0.11	0	0	4.23	yes	no
	c.1151A>G	p.D384G	9:g.128527200A>G	3	mis	E8/16	0.000000	1	0.06	0.42	0.51	-0.02	0.44	0.99	0.7	6.79	no	no
II	c.1422C>A	p.D474E	9:g.128533622C>A	3	mis	E10/16	0.000163	1	0.6	0.54	0.14	-0.98	0.07	0.07	0.05	-0.16	yes	no
	c.1705C>T	p.R569C	9:g.128536413C>T	3	mis	E12/16	0.000227	1	0	0.79	0.61	0.4	0.76	0.96	0.48	2.37	yes	no
	c.1808G>A	p.R603H	9:g.128538017G>A	3	mis	E13/16	0.000059	1	0.01	0.38	0.56	0.09	0.62	1	0.98	8.11	yes	no
	c.2069T>G	p.F690C	9:g.128541142T>G	3	mis	E16/16	0.000146	3	0.17	0.52	0.34	-0.33	0.3	0.99	0.61	1.03	yes	no
	c.2081C>G	p.S694C	9:g.128541154C>G	3	mis	E16/16	0.000000	1	0.01	0.54	0.46	0	0.31	0.77	0.4	3.49	yes	no
HNRNPA1	c.-1C>T	-	12:g.54280807C>T	3	5prUTR	E1/11	0.000115	1	0	0	0	0	0	0	0	0	yes	no
	c.549G>C	p.K183N	12:g.54282452G>C	3	mis	E5/11	0.000000	1	0.02	0.85	0.9	0.8	0.55	0.96	0.7	0.57	no	no
	c.635G>A	p.G212E	12:g.54282624G>A	3	mis	E6/11	0.000024	1	0.01	0.69	0.86	0.84	0.71	0.31	0.05	4.47	yes	no
	c.752-6T>C	-	12:g.54283073T>C	3	sp_re&int	I7/10	0.000000	1	0	0	0	0	0	0	0	0	no	no
NEK1	c.89A>G	p.Y30C	4:g.169602542T>C	3	mis	E3/36	0.000751	1	0.12	0.65	0.48	-0.14	0.57	1	1	5.14	yes	no
	c.214+6A>G	-	4:g.169602002T>C	3	sp_re&int	I4/35	0.002331	1	0	0	0	0	0	0	0	0	yes	no
	c.215-5dupT	-	4:g.169599205dup	3	sp_re&int	I4/35	0.000000	1	0	0	0	0	0	0	0	0	no	no
	c.251A>T	p.E84V	4:g.169599161T>A	3	mis	E5/36	0.000183	1	0	0.76	0.22	-0.64	0.56	1	1	7.69	yes	no
	c.292G>A	p.V98I	4:g.169599120C>T	3	mis	E5/36	0.000778	1	0.64	0.32	0.1	-1	0.11	0.04	0.03	-0.05	yes	no
	c.317T>G	p.L106W	4:g.169590805A>C	3	mis	E6/36	0.000181	1	0	0.73	0.11	-1	0.36	1	0.99	8.91	yes	no
	c.473A>T	p.E158V	4:g.169588727T>A	3	mis	E8/36	0.000293	1	0	0.71	0.37	-0.27	0.5	1	0.99	7.69	no	no
	c.609T>G	p.F203L	4:g.169585547A>C	3	mis&sp_re	E10/36	0.000000	1	0.01	0.92	0.2	-0.73	0.62	1	1	4.42	no	no
	c.694C>T	p.R232C	4:g.169585462G>A	3	mis	E10/36	0.000100	1	0	0.66	0.48	-0.04	0.73	1	1	9.86	yes	no
	c.782G>A	p.R261H	4:g.169585374C>T	3	mis	E10/36	0.008876	33	0.01	0.68	0.12	-1.06	0.26	1	1	7.8	yes	no
	c.842C>T	p.S281L	4:g.169580868G>A	3	mis	E11/36	0.000270	1	0.53	0.35	0.04	-1.06	0.04	0.55	0.06	1.75	yes	no
	c.1021G>A	p.A341T	4:g.169562196C>T	3	mis&sp_re	E13/36	0.006345	3	0.04	0.34	0.3	-0.56	0.12	0.13	0.09	1.28	yes	no
	c.1030A>G	p.T344A	4:g.169562187T>C	3	mis	E13/36	0.000100	1	0.45	0.31	0.16	-0.99	0.08	0.01	0.01	1.72	yes	no
	c.1451G>A	p.R484H	4:g.169555831C>T	3	mis	E18/36	0.000463	1	0.4	0.2	0.13	-1.04	0.05	0	0	0.48	yes	no
	c.1634T>C	p.M545T	4:g.169537840A>G	3	mis	E19/36	0.000279	1	0	0.29	0.22	-0.81	0.06	0.09	0.06	3.41	yes	no
	c.1714T>G	p.S572A	4:g.169508804A>C	3	mis	E20/36	0.000008	1	0.64	0.43	0.1	-1.06	0.09	0	0	1.09	yes	no
	c.1750-5T>C	-	4:g.169508336A>G	3	sp_re&int	I20/35	0.000739	1	0	0	0	0	0	0	0	0	yes	no
	c.1793A>G	p.N598S	4:g.169508288T>C	3	mis	E21/36	0.000213	2	0	0.51	0.47	-0.05	0.27	0.97	0.63	6.91	yes	no
	c.1825G>C	p.G609R	4:g.169508256C>G	3	mis	E21/36	0.000000	1	0	0.64	0.59	0.18	0.41	1	0.98	6.81	no	no
	c.1875G>T	p.E625D	4:g.169507751C>A	3	mis	E22/36	0.000018	1	0.13	0.37	0.23	-0.91	0.16	0.01	0.01	-0.29	yes	no
	c.1889G>T	p.R630L	4:g.169507737C>A	3	mis	E22/36	0.000140	1	0.06	0.36	0.27	-0.58	0.08	0.12	0.07	1.18	yes	no
	c.1942A>G	p.K648E	4:g.169507102T>C	3	mis	E23/36	0.000391	2	0.02	0.65	0.47	-0.08	0.28	0.94	0.64	5.92	yes	no
	c.1958G>A	p.R653Q	4:g.169507086C>T	3	mis	E23/36	0.000534	2	0.08	0.43	0.21	-0.46	0.1	0.52	0.1	2.42	yes	no
	c.2045C>T	p.P682L	4:g.169479497G>A	3	mis	E24/36	0.000130	2	0.07	0.31	0.2	-0.86	0.03	0.01	0.02	0.85	yes	no

	c.2060A>G	p.H687R	4:g.169479482T>C	3	mis	E24/36	0.000015	1	0.5	0.29	0.09	-1.03	0.05	0	0	0.15	yes	no
	c.2110G>A	p.V704I	4:g.169479432C>T	3	mis	E24/36	0.000372	1	0.24	0.31	0.14	-1	0.04	0.01	0	1	yes	no
	c.2352T>A	p.D784E	4:g.169477206A>T	3	mis	E26/36	0.000122	1	0.2	0.45	0.35	-0.63	0.25	0.53	0.18	1.24	yes	no
	c.2395G>C	p.D799H	4:g.169477163C>G	3	mis	E26/36	0.001502	1	0.11	0.32	0.41	-0.19	0.17	0.98	0.78	2.34	yes	no
	c.2408T>C	p.L803P	4:g.169477150A>G	3	mis	E26/36	0.000200	1	0.01	0.38	0.56	-0.02	0.37	1	0.96	3.98	yes	no
	c.2500A>T	p.S834C	4:g.169463330T>A	3	mis	E27/36	0.000846	1	0	0.44	0.43	-0.41	0.2	1	0.84	3	yes	no
	c.3193A>G	p.T1065A	4:g.169424582T>C	3	mis	E31/36	0.001543	1	0.1	0.62	0.32	-0.48	0.31	0.73	0.4	4.16	yes	no
	c.3343A>G	p.I1115V	4:g.169406627T>C	3	mis	E32/36	0.000000	1	0.6	0.31	0.05	-1.02	0.02	0	0	0.54	no	no
	c.3583+4C>T	-	4:g.169401648G>A	3	sp_re&int	I33/35	0.000029	1	0	0	0	0	0	0	0	0	yes	no
	c.3583+8A>C	-	4:g.169401644T>G	3	sp_re&int	I33/35	0.001062	2	0	0	0	0	0	0	0	0	yes	no
	c.3607A>T	p.S1203C	4:g.169400628T>A	3	mis	E34/36	0.000511	1	0	0.57	0.29	-0.41	0.14	1	0.96	3.55	yes	no
	c.3622G>A	p.D1208N	4:g.169400613C>T	3	mis	E34/36	0.002567	1	0.04	0.65	0.32	-0.42	0.24	1	1	7.43	yes	no
	c.3637C>A	p.H1213N	4:g.169400598G>T	3	mis	E34/36	0.001502	1	0.04	0.65	0.29	-0.52	0.16	0.99	0.84	9.43	yes	no
	c.3823A>G	p.M1275V	4:g.169400249T>C	3	mis	E35/36	0.000000	1	0	0.72	0.51	-0.04	0.5	1	0.99	7.54	no	no
OPTN	c.404A>C	p.E135A	10:g.13112487A>C	3	mis	E5/15	0.001040	1	0.32	0.32	0.5	-0.6	0.17	0.04	0.02	1.7	yes	no
	c.425A>C	p.Q142P	10:g.13112508A>C	3	mis	E5/15	0.000088	1	0.01	0.41	0.73	0.69	0.41	0.96	0.63	5.06	yes	no
	c.844A>T	p.T282S	10:g.13122449A>T	3	mis	E8/15	0.000000	1	0.34	0.31	0.32	-0.76	0.13	0.13	0.06	-0.33	yes	no
	c.875C>T	p.P292L	10:g.13122480C>T	3	mis	E8/15	0.000100	1	0.32	0.24	0.1	-1.01	0.26	0	0	0.2	yes	no
	c.883-10C>T	-	10:g.13123985C>T	3	int	I8/14	0.000299	1	0	0	0	0	0	0	0	0	yes	no
	c.941A>T	p.Q314L	10:g.13124053A>T	3	mis	E9/15	0.001873	2	0	0.55	0.79	0.68	0.74	1	0.96	7.46	yes	no
	c.1414T>C	p.C472R	10:g.13132079T>C	3	mis	E13/15	0.000000	1	0	0.66	0.65	0.07	0.5	1	0.96	7.15	yes	no
	c.1509T>G	p.N503K	10:g.13132174T>G	3	mis	E13/15	0.000375	2	0.11	0.55	0.44	-0.62	0.31	0.06	0.04	-0.62	yes	no
	c.1679A>G	p.E560G	10:g.13136811A>G	3	mis	E15/15	0.000000	1	0.01	0.45	0.65	-0.12	0.36	0.98	0.72	4.1	no	no
PFN1	c.350_351delinsGT	p.E117G	17:g.4945972_4945973delinsAC	3	mis	E3/3	0.000000	5	0	0	0	0	0	0	0	0	yes	no
SOD1	c.331C>T	p.H111Y	21:g.31667349C>T	3	mis	E4/5	0.000000	1	0.17	0.3	0.9	0.8	0.19	0	0	-1.29	yes	no
SQSTM1	c.98C>T	p.A33V	5:g.179821034C>T	3	mis	E1/8	0.016260	2	1	0.78	0.24	-0.87	0.05	0.01	0	2.76	yes	no
	c.205+7G>A	-	5:g.179821148G>A	3	sp_re&int	I1/7	0.000146	1	0	0	0	0	0	0	0	0	yes	no
	c.287G>A	p.R96Q	5:g.179823039G>A	3	mis	E2/8	0.001096	1	0	0.58	0.78	0.71	0.44	0	0	9.77	yes	no
	c.316C>G	p.R106G	5:g.179823872C>G	3	mis	E3/8	0.000000	1	0.07	0.44	0.3	-0.46	0.07	0	0	3.18	yes	no
	c.335C>T	p.P112L	5:g.179823891C>T	3	mis	E3/8	0.000146	1	0.13	0.33	0.12	-1	0.1	0	0	0.37	yes	no
	c.431T>A	p.V144D	5:g.179823987T>A	3	mis	E3/8	0.000015	1	0	0.6	0.77	0.68	0.76	0	0	7.83	yes	no
	c.508A>G	p.S170G	5:g.179824064A>G	3	mis	E3/8	0.000751	1	0.27	0.29	0.38	-0.67	0.21	0	0	4.67	yes	no
	c.547C>T	p.R183C	5:g.179824197C>T	3	mis	E4/8	0.001447	1	0	0.52	0.64	0.36	0.8	0	0	2.71	yes	no
	c.1045T>A	p.S349T	5:g.179833662T>A	3	mis	E7/8	0.000009	1	0	0.81	0.76	0.47	0.66	0.97	0.56	7.45	yes	no
	c.1207T>C	p.S403P	5:g.179836477T>C	3	mis	E8/8	0.000000	1	0	0.76	0.8	0.77	0.84	1	0.97	7.99	no	no
TARDBP	c.976G>A	p.A326T	1:g.11022385G>A	3	mis	E6/6	0.000000	1	0.07	0.79	0.91	0.87	0.65	1	0.98	9.04	no	no
	c.1187G>A	p.G396D	1:g.11022596G>A	3	mis	E6/6	0.000000	1	0.03	0.83	0.81	0.75	0.68	1	0.99	9.11	no	no
TBK1	c.217A>G	p.I73V	12:g.64460318A>G	3	mis	E3/21	0.001266	1	1	0.48	0.11	-1	0.09	0	0.01	5.04	yes	no
	c.229-5_229-4dupTT	-	12:g.64464329_64464330dup	3	sp_re&int	I3/20	0.000000	1	0	0	0	0	0	0	0	0	no	no
	c.236_238delCAA	p.TR79_TR80delinsR	12:g.64464341_64464343del	3	inf_del	E4/21	0.000000	1	0	0	0	0	0	0	0	0	no	no
	c.314A>G	p.Y105C	12:g.64464419A>G	3	mis	E4/21	0.000375	1	0	0.83	0.29	-0.55	0.3	0.94	0.7	1.82	yes	no
	c.350G>A	p.R117Q	12:g.64464455G>A	3	mis	E4/21	0.000641	1	0.96	0.51	0.06	-1.08	0.04	0	0	3.76	yes	no
	c.385A>G	p.N129D	12:g.64466927A>G	3	mis	E5/21	0.000000	1	0.02	0.87	0.43	0.09	0.43	0.91	0.49	5.37	yes	no
	c.511G>A	p.V171I	12:g.64467053G>A	3	mis	E5/21	0.000000	1	0.18	0.79	0.37	-0.45	0.2	0.99	0.99	5.74	no	no
	c.521A>G	p.Y174C	12:g.64467063A>G	3	mis	E5/21	0.000008	1	0	0.9	0.24	-0.65	0.59	1	1	9.19	yes	no
	c.562G>A	p.A188T	12:g.64474251G>A	3	mis	E6/21	0.000000	1	0	0.89	0.56	0.06	0.55	1	1	9.52	no	no
	c.563C>A	p.A188E	12:g.64474252C>A	3	mis	E6/21	0.000000	1	0.02	0.91	0.58	0.15	0.61	1	1	7.51	no	no
	c.818T>G	p.L273R	12:g.64481847T>G	3	mis	E8/21	0.000000	1	0	0.75	0.36	-0.04	0.76	1	1	7.52	no	no
	c.871A>G	p.K291E	12:g.64481900A>G	3	mis	E8/21	0.000427	5	0.03	0.84	0.22	-0.74	0.44	1	0.99	8.77	yes	no
	c.1063G>A	p.E355K	12:g.64484373G>A	3	mis	E9/21	0.000000	1	0.01	0.7	0.38	-0.45	0.44	1	0.97	8.02	yes	no
	c.1190T>C	p.I397T	12:g.64485455T>C	3	mis&sp_re	E10/21	0.001502	1	0.11	0.65	0.14	-0.96	0.1	0.04	0.02	5.02	yes	yes
	c.1323G>T	p.K441N	12:g.64486000G>T	3	mis	E11/21	0.000000	1	0.03	0.68	0.14	-1.03	0.11	0.75	0.25	1.39	yes	no
	c.1644-5_1644-2del	-	12:g.64495694_64495697del	3	sp_ac&sp_re&int	I14/20	0.000000	1	0	0	0	0	0	0	0	0	no	yes
	c.1760+4_1760+7del	-	12:g.64496410_64496413del	3	sp_re&int	I16/20	0.000000	1	0	0	0	0	0	0	0	0	no	no
	c.1959+5G>T	-	12:g.64497264G>T	3	sp_re&int	I18/20	0.000000	1	0	0	0	0	0	0	0	0	no	no
	c.2075A>C	p.K692T	12:g.64497976A>C	3	mis	E20/21	0.000000	1	0.01	0.81	0.35	-0.46	0.33	1	0.99	7.24	no	no
	c.2125_2133del	p.HIL709del	12:g.64498026_64498034del	3	inf_del	E20/21	0.000000	1	0	0	0	0	0	0	0	0	no	no
	c.2177T>C	p.V726A	12:g.64501368T>C	3	mis	E21/21	0.001092	1	0.18	0.71	0.25	-0.64	0.3	0.92	0.86	4.35	yes	no
TUBA4A	c.316G>T	p.G106C	2:g.219251624C>A	3	mis	E3/4	0.000000	1	0	0.94	0.84	0.97	0.95	1	1	7.82	no	no
	c.376-6T>G	-	2:g.219251329A>C	3	sp_re&int	I3/3	0.000015	1	0	0	0	0	0	0	0	0	yes	no
	c.1291G>A	p.D431N	2:g.219250408C>T	3	mis	E4/4	0.000000	1	0	0.86	0.8	0.8	0.72	0.91	0.92	7.9	no	no
UBQLN2	c.383C>T	p.T128I	X:g.56564256C>T	3	mis	E1/1	0.000000	1	0.17	0.4	0.44	0	0.35	0.8	0.37	1.67	no	no
	c.1019G>T	p.S340I	X:g.56564892G>T	3	mis	E1/1	0.000037	1	0.03	0.42	0.43	-0.36	0.38	0.87	0.48	0.59	yes	no

	c.1057A>T	p.T353S	X:g.56564930A>T	3	mis	E1/1	0.000512	1	1	0.48	0.13	-0.93	0.28	0.02	0.01	4.1	yes	no
	c.1318C>A	p.P440T	X:g.56565191C>A	3	mis	E1/1	0.000038	1	0	0.7	0.82	0.82	0.69	1	1	5.98	yes	no
	c.1420C>G	p.P474A	X:g.56565293C>G	3	mis	E1/1	0.000523	1	0.44	0.75	0.62	0.33	0.64	0.88	0.44	7.36	yes	no
	c.1624G>T	p.A542S	X:g.56565497G>T	3	mis	E1/1	0.000100	1	0.79	0.41	0.49	-0.34	0.17	0.05	0.01	1.91	yes	no
	c.1835A>G	p.N612S	X:g.56565708A>G	3	mis	E1/1	0.000000	1	0.07	0.72	0.12	-1	0.23	0.99	0.98	5.35	no	no
VAPB	c.149G>A	p.R50H	20:g.58418301G>A	3	mis	E2/6	0.000035	1	0.02	0.87	0.5	-0.04	0.5	0.91	0.25	8.15	yes	no
	c.310G>A	p.A104T	20:g.58434700G>A	3	mis	E3/6	0.000008	1	0.23	0.67	0.23	-0.83	0.28	0.93	0.6	10	yes	no
	c.512A>T	p.E171V	20:g.58441022A>T	3	mis	E5/6	0.000000	1	0	0.52	0.27	-0.44	0.43	1	1	8.95	yes	no
VCP	c.41C>T	p.T14I	9:g.35068339G>A	3	mis	E2/17	0.000065	1	0.03	0.91	0.82	0.71	0.67	0.42	0.07	9.85	yes	no

Supplemental Table 5 - B Overview of variants of uncertain significance in ALS genes (tenuous evidence)

Gene	c.HGVS	p.HGVS	g.HGVS	ACMG Class (C)	SNPEff / VEP consensus effect	Transcript	MaxFreq Database	Frequency (no of patients)	SIFT	PrimateAI	MetaLR score	MetaSVM score	REVEL score	Polyphen2 HDIV score	Polyphen2 HVAR score	phyloP100way vertebrate	Known Variant	Null variant
ALS2	c.331G>A	p.V111I	2:g.201761663C>T	3	mis	E4/34	0.001293	1	0.4	0.22	0.36	-0.75	0.09	0.02	0.05	1.23	yes	no
	c.853A>G	p.R285G	2:g.201761141T>C	3	mis	E4/34	0.004065	1	0.41	0.19	0.08	-1.03	0.04	0.00	0.00	0.87	yes	no
	c.949_960del	p.AMS317_MSS320del	2:g.201761035_201761046del	3	inf_del	E4/34	0.000000	1	0	0.00	0.00	0.00	0.00	0.00	0.00	0.00	no	no
	c.1049G>A	p.R350Q	2:g.201760945C>T	3	mis	E4/34	0.000200	1	0.8	0.17	0.06	-0.99	0.02	0.00	0.00	-0.09	yes	no
	c.1115C>G	p.P372R	2:g.201757758G>C	3	mis	E5/34	0.004065	3	0.3	0.37	0.09	-1.07	0.12	0.53	0.22	1.88	yes	no
	c.1130C>A	p.A377E	2:g.201757743G>T	3	mis	E5/34	0.000000	1	0.5	0.34	0.07	-1.03	0.06	0.00	0.00	1.42	no	no
	c.1265T>C	p.M422T	2:g.201757608A>G	3	mis	E5/34	0.001721	1	0.14	0.88	0.21	-0.76	0.23	0.72	0.16	8.46	yes	no
	c.1471+10T>A	-	2:g.201757392A>T	3	int	I5/33	0.000000	1	0	0.00	0.00	0.00	0.00	0.00	0.00	0.00	no	no
	c.1550C>G	p.A517G	2:g.201754593G>C	3	mis	E6/34	0.001988	1	0.03	0.69	0.29	-0.54	0.22	0.94	0.50	9.34	yes	no
	c.1718C>T	p.A573V	2:g.201753165G>A	3	mis	E7/34	0.000033	1	0.04	0.68	0.72	0.49	0.78	0.44	0.45	8.90	yes	no
	c.1737+3A>G	-	2:g.201753143T>C	3	sp_re&sp_do&int	I7/33	0.000065	1	0	0.00	0.00	0.00	0.00	0.00	0.00	0.00	yes	no
	c.2048T>C	p.L683S	2:g.201744380A>G	3	mis	E10/34	0.000146	1	0	0.57	0.65	0.42	0.84	1.00	0.99	8.32	yes	no
	c.2352-6C>T	-	2:g.201738741G>A	3	sp_re&int&sp_tr	I11/33	0.000000	1	0	0.00	0.00	0.00	0.00	0.00	0.00	0.00	no	no
	c.2479A>T	p.T827S	2:g.201733377T>A	3	mis	E13/34	0.007241	3	0.58	0.32	0.07	-1.02	0.08	0.00	0.01	0.65	yes	no
	c.2632C>T	p.L878F	2:g.201729132G>A	3	mis	E14/34	0.002404	1	0	0.62	0.34	-0.39	0.30	0.81	0.76	3.59	yes	no
	c.2777A>C	p.Q926P	2:g.201728576T>G	3	mis	E15/34	0.000000	1	0.01	0.81	0.62	0.30	0.47	1.00	0.96	5.85	yes	no
	c.3206G>A	p.G1069E	2:g.201726526C>T	3	mis	E19/34	0.022059	1	0	0.69	0.72	0.51	0.84	1.00	1.00	7.50	yes	no
	c.3442C>T	p.P1148S	2:g.201724365G>A	3	mis	E21/34	0.000955	1	0.46	0.76	0.06	-1.04	0.22	0.59	0.15	6.74	yes	no
	c.3518A>C	p.E1173A	2:g.201723436T>G	3	mis	E22/34	0.000000	1	0	0.80	0.16	-0.91	0.34	1.00	0.90	8.02	yes	no
	c.3692T>A	p.L1231H	2:g.201723053A>T	3	mis	E23/34	0.000015	1	0	0.67	0.64	0.24	0.67	1.00	1.00	9.33	yes	no
	c.3814G>A	p.D1272N	2:g.201718099C>T	3	mis	E24/34	0.001988	1	0.12	0.49	0.12	-1.03	0.06	0.04	0.01	7.36	yes	no
	c.4871T>C	p.I1624T	2:g.201704186A>G	3	mis	E33/34	0.000400	1	0	0.88	0.32	-0.34	0.77	1.00	0.98	9.27	yes	no
	c.4957C>T	p.R1653C	2:g.201701868G>A	3	mis	E34/34	0.000319	2	0.02	0.47	0.09	-1.00	0.35	0.98	0.85	7.62	yes	no
	c.4966C>T	p.L1656F	2:g.201701859G>A	3	mis	E34/34	0.000000	1	0.01	0.68	0.27	-0.55	0.20	0.97	0.94	4.38	no	no
DCTN1	c.59C>T	p.A20V	2:g.74378220G>A	3	mis	E2/32	0.000164	1	0.44	0.54	0.16	-0.91	0.15	0.00	0.00	3.80	yes	no
	c.460C>T	p.R154C	2:g.74371722G>A	3	mis	E8/32	0.000196	2	0	0.68	0.41	-0.09	0.34	1.00	0.45	8.36	yes	no
	c.577_578delinsAA	p.A193K	2:g.74371604_74371605delinsTT	3	mis	E8/32	0.000000	1	0.04	0.00	0.00	0.00	0.00	0.00	0.00	0.00	no	no
	c.673C>T	p.R225W	2:g.74371149G>A	3	mis	E9/32	0.001475	1	0	0.76	0.53	0.10	0.46	0.99	0.35	2.65	yes	no
	c.1288-10C>G	-	2:g.74370079G>C	3	int&sp_tr	I12/31	0.000000	1	0	0.00	0.00	0.00	0.00	0.00	0.00	0.00	yes	no
	c.1337G>A	p.R446Q	2:g.74370020C>T	3	mis	E13/32	0.000000	1	0.03	0.65	0.54	-0.06	0.35	1.00	0.95	7.72	no	no
	c.1480G>A	p.A494T	2:g.74369404C>T	3	mis	E14/32	0.001135	1	0.15	0.67	0.49	-0.27	0.25	1.00	0.83	7.72	yes	no
	c.1867C>T	p.R623W	2:g.74368119G>A	3	mis	E17/32	0.000145	1	0.01	0.80	0.42	-0.20	0.40	0.99	0.76	3.77	yes	no
	c.2006G>A	p.R669H	2:g.74367980C>T	3	mis	E17/32	0.000321	2	0.09	0.67	0.37	-0.33	0.32	0.97	0.52	3.79	yes	no
	c.2020C>T	p.L674F	2:g.74367860G>A	3	mis	E18/32	0.000116	1	0	0.65	0.75	0.54	0.71	1.00	1.00	4.52	yes	no
	c.2318G>T	p.G773V	2:g.74366931C>A	3	mis&sp_re	E21/32	0.001535	1	0.22	0.50	0.20	-0.81	0.25	0.00	0.00	1.75	yes	yes
	c.2377G>A	p.D793N	2:g.74366872C>T	3	mis	E21/32	0.000000	1	0.01	0.88	0.66	0.13	0.37	1.00	0.99	7.29	no	no
	c.2633A>G	p.Y878C	2:g.74366371T>C	3	mis	E23/32	0.000213	1	0	0.73	0.60	0.26	0.68	1.00	0.99	7.62	yes	no
	c.2642C>T	p.P881L	2:g.74366362G>A	3	mis	E23/32	0.000000	1	0.26	0.47	0.20	-0.83	0.20	0.02	0.00	4.53	no	no
	c.2746C>T	p.R916W	2:g.74366258G>A	3	mis	E23/32	0.000321	1	0.01	0.76	0.40	-0.24	0.49	0.99	0.55	1.13	yes	no
	c.3097C>G	p.L1033V	2:g.74365174G>C	3	mis	E26/32	0.000000	1	0	0.68	0.58	0.20	0.49	0.99	0.95	7.59	yes	no
	c.3128G>A	p.R1043H	2:g.74365143C>T	3	mis	E26/32	0.001721	2	0.03	0.51	0.53	0.11	0.38	0.98	0.28	3.60	yes	no
	c.3131C>T	p.T1044M	2:g.74365140G>A	3	mis	E26/32	0.000044	1	0.01	0.56	0.49	-0.01	0.35	0.66	0.06	4.47	yes	no
	c.3134T>C	p.I1045T	2:g.74365137A>G	3	mis	E26/32	0.000008	2	0.18	0.52	0.21	-0.81	0.17	0.00	0.00	4.59	yes	no
	c.3137A>G	p.E1046G	2:g.74365134T>C	3	mis	E26/32	0.000539	1	0.04	0.59	0.38	-0.41	0.40	0.73	0.24	7.64	yes	no
	c.3448C>T	p.R1150C	2:g.74363075G>A	3	mis	E29/32	0.000024	1	0	0.58	0.63	0.34	0.55	1.00	0.99	4.11	yes	no
	c.3537G>C	p.K1179N	2:g.74362722C>G	3	mis	E30/32	0.001475	1	0.46	0.56	0.19	-0.72	0.10	0.20	0.15	0.46	yes	no
	c.3662C>G	p.T1221S	2:g.74362089G>C	3	mis	E31/32	0.000000	1	0.85	0.51	0.16	-0.89	0.17	0.00	0.00	5.92	no	no
	c.3733A>G	p.M1245V	2:g.74361603T>C	3	mis	E32/32	0.000262	1	1	0.31	0.10	-0.98	0.09	0.00	0.00	0.39	yes	no

GRN	c.22G>A	p.V8M	17:g.44349186G>A	3	mis	E2/13	0.000100	2	0.07	0.49	0.29	-0.78	0.17	0.89	0.41	0.89	yes	no
	c.53C>T	p.T18M	17:g.44349217C>T	3	mis	E2/13	0.000640	1	0.06	0.43	0.38	-0.68	0.32	1.00	0.74	0.29	yes	no
	c.99C>A	p.D33E	17:g.44349263C>A	3	mis	E2/13	0.002600	4	0.53	0.37	0.18	-0.86	0.12	0.74	0.21	-0.12	yes	no
	c.128G>A	p.R43H	17:g.44349292G>A	3	mis	E2/13	0.000344	1	0.04	0.27	0.12	-1.02	0.06	0.20	0.05	-0.39	yes	no
	c.173G>A	p.G58D	17:g.44349460G>A	3	mis	E3/13	0.000000	1	0.23	0.38	0.13	-0.97	0.10	0.48	0.09	0.53	no	no
	c.229G>A	p.V77I	17:g.44349516G>A	3	mis	E3/13	0.002950	2	0.42	0.30	0.17	-0.97	0.07	0.06	0.02	-0.83	yes	no
	c.302G>A	p.R101Q	17:g.44349704G>A	3	mis	E4/13	0.000600	1	0.35	0.21	0.11	-0.99	0.04	0.32	0.10	-0.66	yes	no
	c.322G>A	p.D108N	17:g.44349724G>A	3	mis	E4/13	0.000000	1	0.09	0.37	0.26	-0.86	0.16	0.08	0.14	1.51	no	no
	c.350-3C>T	-	17:g.44350225C>T	3	sp_re&sp_tr&int	I4/12	0.000023	1	0	0.00	0.00	0.00	0.00	0.00	0.00	0.00	yes	no
	c.415T>C	p.C139R	17:g.44350293T>C	3	mis	E5/13	0.000489	1	0	0.65	0.86	0.93	0.84	1.00	1.00	5.06	yes	no
	c.542C>T	p.P181L	17:g.44350521C>T	3	mis	E6/13	0.000015	1	0.12	0.29	0.24	-0.86	0.17	0.25	0.05	1.53	no	no
	c.836-3C>T	-	17:g.44351360C>T	3	sp_tr&sp_re&int	I8/12	0.000375	1	0	0.00	0.00	0.00	0.00	0.00	0.00	0.00	yes	no
	c.1180-7C>G	-	17:g.44352008C>G	3	sp_re&sp_tr&int	I10/12	0.000140	1	0	0.00	0.00	0.00	0.00	0.00	0.00	0.00	yes	no
	c.1648G>A	p.V550I	17:g.44352664G>A	3	mis	E13/13	0.001020	1	0.27	0.25	0.32	-0.85	0.29	0.00	0.01	-1.35	yes	no
HNRNPA2B1	c.7-9dupT	-	7:g.26197884dup	3	sp_tr&int&nmd	I1/12	0.023525	2	0	0.00	0.00	0.00	0.00	0.00	0.00	0.00	yes	no
	c.43-8C>T	-	7:g.26197740G>A	3	sp_re&sp_tr&int&nmd	I2/12	0.000060	1	0	0.00	0.00	0.00	0.00	0.00	0.00	0.00	yes	no
	c.511+9C>G	-	7:g.26196798G>C	3	int&nmd	I5/12	0.000191	1	0	0.00	0.00	0.00	0.00	0.00	0.00	0.00	yes	no
	c.512-8C>T	-	7:g.26196666G>A	3	sp_re&sp_tr&int&nmd	I5/12	0.000015	1	0	0.00	0.00	0.00	0.00	0.00	0.00	0.00	yes	no
	c.1058A>G	p.Y353C	7:g.26192520T>C	3	noncod_ex&mis&nmd	E11/13	0.000000	1	0	0.78	0.49	0.18	0.00	1.00	0.56	6.31	no	no
MAPT	c.89C>T	p.T30I	17:g.45962426C>T	3	mis	E1/14	0.000146	1	0	0.45	0.15	-0.94	0.11	0.85	0.47	1.89	yes	no
	c.293C>G	p.P98R	17:g.45974457C>G	3	mis	E3/14	0.000000	1	0	0.55	0.17	-0.94	0.16	1.00	1.00	3.28	yes	no
	c.308-3C>T	-	17:g.45978372C>T	3	sp_re&sp_tr&int	I3/13	0.000000	1	0	0.00	0.00	0.00	0.00	0.00	0.00	0.00	no	no
	c.320G>T	p.G107V	17:g.45978387G>T	3	mis	E4/14	0.000427	1	0	0.61	0.27	-0.58	0.15	1.00	1.00	5.92	yes	no
	c.530A>T	p.D177V	17:g.45983334A>T	3	mis	E5/14	0.002183	1	0.09	0.38	0.04	-1.05	0.08	0.88	0.31	0.06	yes	no
	c.550G>A	p.A184T	17:g.45983354G>A	3	mis	E5/14	0.003165	1	0.27	0.29	0.02	-0.99	0.02	0.61	0.09	0.02	yes	no
	c.664C>A	p.R222S	17:g.45983468C>A	3	mis	E5/14	0.016260	1	0.03	0.32	0.04	-1.09	0.08	0.83	0.15	2.80	yes	no
	c.683C>G	p.S228C	17:g.45983487C>G	3	mis	E5/14	0.000391	1	0.06	0.36	0.05	-1.14	0.09	0.99	0.71	0.36	yes	no
	c.782C>T	p.A261V	17:g.45983586C>T	3	mis	E5/14	0.000321	1	0.18	0.26	0.02	-1.00	0.02	0.74	0.09	-0.49	yes	no
	c.878A>G	p.K293R	17:g.45983682A>G	3	mis	E5/14	0.000164	1	0.06	0.34	0.01	-0.93	0.02	0.00	0.00	0.46	yes	no
	c.1139G>A	p.S380N	17:g.45987052G>A	3	mis	E6/14	0.002612	1	0.01	0.47	0.09	-1.02	0.02	0.65	0.21	3.54	yes	no
	c.1228C>T	p.L410F	17:g.45989923C>T	3	mis	E7/14	0.000431	1	0.05	0.35	0.13	-0.99	0.04	0.97	0.62	3.46	yes	no
	c.1280C>T	p.S427F	17:g.45989975C>T	3	mis	E7/14	0.007353	1	0.02	0.47	0.24	-0.64	0.15	1.00	0.99	2.40	yes	no
	c.1537C>A	p.P513T	17:g.45993953C>A	3	mis	E9/14	0.004545	2	0.14	0.43	0.03	-1.10	0.08	0.91	0.68	0.63	yes	no
	c.2159A>G	p.K720R	17:g.46023999A>G	3	mis	E14/14	0.000000	1	0.33	0.69	0.04	-1.00	0.16	1.00	0.99	3.56	no	no
MATR3	c.912+8A>C	-	5:g.139308335A>C	3	sp_re&int	I2/14	0.000000	1	0	0.00	0.00	0.00	0.00	0.00	0.00	0.00	no	no
	c.1105G>A	p.A369T	5:g.139316164G>A	3	mis	E5/15	0.000000	1	0.37	0.63	0.22	-0.60	0.07	0.03	0.01	4.86	no	no
	c.1130-6A>G	-	5:g.139317047A>G	3	sp_re&sp_tr&int	I5/14	0.000000	1	0	0.00	0.00	0.00	0.00	0.00	0.00	0.00	no	no
	c.1464T>A	p.F488L	5:g.139319363T>A	3	mis	E9/15	0.000431	1	0.66	0.82	0.06	-1.00	0.17	0.02	0.02	0.30	yes	no
	c.1778+1_1778+91del	-	5:g.139322507_139322597del	3	sp_ac&sp_do&int	I11/14	0.000000	14	0	0.00	0.00	0.00	0.00	0.00	0.00	0.00	no	yes
	c.1996G>A	p.A666T	5:g.139322815G>A	3	mis	E12/15	0.000054	2	0.09	0.68	0.50	0.08	0.36	0.46	0.10	4.95	yes	no
	c.2035A>G	p.T679A	5:g.139322854A>G	3	mis	E12/15	0.000066	1	0.87	0.54	0.12	-0.96	0.15	0.00	0.00	0.95	yes	no
	c.2138A>C	p.K713T	5:g.139322957A>C	3	mis	E12/15	0.000009	1	0.11	0.70	0.47	-0.05	0.24	1.00	0.98	2.81	yes	no
	c.2148+3A>G	-	5:g.139322970A>G	3	sp_re&sp_do&int	I12/14	0.019300	1	0	0.00	0.00	0.00	0.00	0.00	0.00	0.00	yes	no
	c.2301T>A	p.D767E	5:g.139325592T>A	3	mis	E13/15	0.000044	1	0.79	0.54	0.16	-0.96	0.14	0.00	0.00	0.38	yes	no
	c.2360A>G	p.N787S	5:g.139325651A>G	3	mis	E13/15	0.003897	1	0.07	0.60	0.23	-0.57	0.21	0.38	0.16	4.65	no	no
	c.2533A>G	p.K845E	5:g.139329384A>G	3	mis	E15/15	0.000059	1	0.09	0.70	0.42	-0.19	0.20	0.01	0.01	5.67	yes	no
NEFH	c.1148G>A	p.R383Q	22:g.29485787G>A	3	mis	E3/4	0.000227	1	0.08	0.79	0.74	0.56	0.67	0.00	0.00	5.74	yes	no
	c.1346A>G	p.K449R	22:g.29488986A>G	3	mis	E4/4	0.000062	1	0	0.55	0.54	0.08	0.46	0.00	0.00	6.71	yes	no
	c.1510_1530del	p.PPA504_EAA510del	22:g.29489150_29489170del	3	inf_del	E4/4	0.000164	1	0	0.00	0.00	0.00	0.00	0.00	0.00	0.00	yes	no
	c.1920_1943del	p.SPT640_AKS648delinsS	22:g.29489560_29489583del	3	inf_del	E4/4	0.000000	14	0	0.00	0.00	0.00	0.00	0.00	0.00	0.00	no	no
	c.2114C>T	p.P705L	22:g.29489754C>T	3	mis	E4/4	0.000000	1	0	0.42	0.73	0.57	0.50	0.00	0.00	1.05	no	no
	c.2160G>T	p.E720D	22:g.29489800G>T	3	mis	E4/4	0.000000	1	0.01	0.31	0.63	-0.47	0.33	0.00	0.00	-0.68	no	no
	c.2214_2219delinsTCCAAC	p.SPV738_SPV740delinsSPT	22:g.29489854_29489859delinsTCCAAC	3	mis	E4/4	0.000000	1	0	0.00	0.00	0.00	0.00	0.00	0.00	0.00	no	no
	c.2474A>G	p.K825R	22:g.29490114A>G	3	mis	E4/4	0.000000	1	0	0.42	0.54	0.20	0.41	0.00	0.00	2.13	no	no
	c.2708T>C	p.V903A	22:g.29490348T>C	3	mis	E4/4	0.000000	1	1	0.29	0.06	-0.94	0.13	0.00	0.00	1.04	no	no
	c.2812G>A	p.A938T	22:g.29490452G>A	3	mis	E4/4	0.000000	1	0.02	0.36	0.56	-0.11	0.23	0.00	0.00	1.23	no	no
	c.3046G>A	p.A1016T	22:g.29490686G>A	3	mis	E4/4	0.000000	1	0.05	0.38	0.51	-0.17	0.26	0.00	0.00	1.95	no	no
PRPH	c.1090G>T	p.E364*	12:g.49297450G>T	3	st_gain	E6/9	0.000100	1	0	0.00	0.00	0.00	0.00	0.00	0.00	9.93	yes	no
	c.1218-4C>T	-	12:g.49297673C>T	3	sp_re&sp_tr&int	I6/8	0.002358	1	0	0.00	0.00	0.00	0.00	0.00	0.00	0.00	yes	no
	c.1376G>A	p.R459H	12:g.49298316G>A	3	mis	E9/9	0.000100	1	0.42	0.21	0.23	-0.93	0.21	0.00	0.00	-1.32	yes	no
SETX	c.71A>G	p.N24S	9:g.132349358T>C	3	mis	E3/26	0.000066	1	0.16	0.26	0.27	-0.90	0.21	0.00	0.00	-0.94	yes	no
	c.502C>T	p.R168W	9:g.132336512G>A	3	mis	E6/26	0.000029	1	0	0.80	0.64	0.40	0.64	1.00	1.00	5.92	yes	no
	c.658A>C	p.K220Q	9:g.132336356T>G	3	mis	E6/26	0.000270	1	0	0.59	0.28	-0.42	0.18	1.00	1.00	4.42	yes	no

	c.710A>G	p.Y237C	9:g.132336304T>C	3	mis	E6/26	0.000270	1	0	0.82	0.33	-0.37	0.55	1.00	1.00	6.19	yes	no
	c.806C>T	p.S269L	9:g.132334640G>A	3	mis	E7/26	0.000140	1	0.02	0.63	0.15	-0.81	0.69	0.09	0.03	2.25	yes	no
	c.991A>G	p.I331V	9:g.132331296T>C	3	mis	E8/26	0.000994	1	0.02	0.48	0.16	-0.80	0.05	0.21	0.06	3.59	yes	no
	c.1260T>A	p.D420E	9:g.132330338A>T	3	mis	E10/26	0.000000	1	0.09	0.72	0.47	-0.28	0.45	0.98	0.84	0.20	no	no
	c.1374T>G	p.F458L	9:g.132330224A>C	3	mis	E10/26	0.000784	2	0	0.72	0.58	-0.07	0.57	1.00	0.99	-0.01	yes	no
	c.1483C>T	p.L495F	9:g.132330115G>A	3	mis	E10/26	0.000140	1	0	0.52	0.62	0.32	0.45	1.00	1.00	7.66	yes	no
	c.1841T>C	p.I614T	9:g.132329757A>G	3	mis	E10/26	0.000016	2	1	0.28	0.18	-0.84	0.12	0.00	0.00	-0.40	yes	no
	c.1919C>G	p.A640G	9:g.132329679G>C	3	mis	E10/26	0.000146	1	0.1	0.25	0.33	-0.81	0.21	0.00	0.00	-0.46	yes	no
	c.2385_2387del	p.IK795_IK796delinsM	9:g.132329211_132329213del	3	inf_del	E10/26	0.001266	1	0	0.00	0.00	0.00	0.00	0.00	0.00	0.00	yes	no
	c.2399G>T	p.R800M	9:g.132329199C>A	3	mis	E10/26	0.000059	1	0	0.29	0.68	-0.26	0.33	0.98	0.62	-0.19	yes	no
	c.2479A>G	p.K827E	9:g.132329119T>C	3	mis	E10/26	0.004065	4	0.05	0.36	0.62	-0.29	0.29	0.73	0.32	0.45	yes	no
	c.2750T>C	p.M917T	9:g.132328848A>G	3	mis	E10/26	0.000724	4	0.21	0.28	0.30	-0.75	0.20	0.01	0.01	0.68	yes	no
	c.2755G>C	p.V919L	9:g.132328843C>G	3	mis	E10/26	0.000539	1	0.51	0.27	0.34	-0.74	0.21	0.00	0.00	-1.86	yes	no
	c.3072_3074dupTGA	p.-1024_-1025ins*	9:g.132328525_132328526insTCA	3	st_gain&inf_ins&st_gain	E10/26	0.000000	2	0	0.00	0.00	0.00	0.00	0.00	0.00	0.00	no	no
	c.3227C>A	p.S1076Y	9:g.132328371G>T	3	mis	E10/26	0.000000	1	0.14	0.38	0.52	-0.55	0.28	0.09	0.02	0.90	no	no
	c.3287A>G	p.H1096R	9:g.132328311T>C	3	mis	E10/26	0.000024	1	0.12	0.31	0.38	-0.69	0.25	0.03	0.01	1.06	yes	no
	c.3809C>T	p.P1270L	9:g.132327789G>A	3	mis	E10/26	0.010985	1	0.04	0.57	0.66	0.28	0.44	0.90	0.15	5.07	yes	no
	c.3826C>G	p.Q1276E	9:g.132327772G>C	3	mis	E10/26	0.026316	3	0	0.33	0.63	-0.24	0.39	0.80	0.27	2.12	yes	no
	c.3899C>T	p.S1300F	9:g.132327699G>A	3	mis	E10/26	0.000000	1	0	0.60	0.89	0.95	0.57	1.00	1.00	7.96	yes	no
	c.3902A>G	p.Q1301R	9:g.132327696T>C	3	mis	E10/26	0.000053	1	0	0.47	0.85	0.90	0.52	1.00	1.00	6.39	yes	no
	c.4225A>T	p.N1409Y	9:g.132327373T>A	3	mis	E10/26	0.000233	1	0.01	0.21	0.47	-0.50	0.69	0.61	0.12	0.55	yes	no
	c.4598A>G	p.E1533G	9:g.132327000T>C	3	mis	E10/26	0.000116	1	0.33	0.27	0.52	-0.78	0.22	0.00	0.00	0.13	yes	no
	c.4612C>T	p.R1538W	9:g.132326986G>A	3	mis	E10/26	0.003882	1	0.21	0.18	0.51	-0.69	0.26	0.00	0.00	0.58	yes	no
	c.4757C>T	p.P1586L	9:g.132326841G>A	3	mis	E10/26	0.000065	2	0.94	0.25	0.19	-0.86	0.26	0.00	0.00	0.94	yes	no
	c.5051C>G	p.S1684C	9:g.132326547G>C	3	mis	E10/26	0.005900	1	0.07	0.25	0.48	-0.45	0.29	0.42	0.14	-0.02	yes	no
	c.5059C>G	p.P1687A	9:g.132326539G>C	3	mis	E10/26	0.000227	1	0.15	0.27	0.49	-0.63	0.13	0.02	0.02	1.36	yes	no
	c.5299C>G	p.P1767A	9:g.132311832G>C	3	mis	E11/26	0.000000	1	0.22	0.41	0.78	0.44	0.39	0.00	0.00	2.71	no	no
	c.5322G>T	p.Q1774H	9:g.132311809C>A	3	mis	E11/26	0.000375	1	0.52	0.30	0.67	-0.38	0.23	0.00	0.00	0.11	yes	no
	c.5374G>A	p.V1792I	9:g.132311757C>T	3	mis&sp_re	E11/26	0.000480	1	0.26	0.37	0.54	-0.55	0.19	0.00	0.00	1.14	yes	yes
	c.5404C>A	p.Q1802K	9:g.132300774G>T	3	mis	E12/26	0.000000	1	0	0.53	0.85	0.86	0.58	0.00	0.00	6.47	no	no
	c.5929C>G	p.L1977V	9:g.132296907G>C	3	mis	E14/26	0.000000	1	0	0.61	0.41	-0.39	0.40	0.00	0.00	1.51	yes	no
	c.5949+5G>A	-	9:g.132296882C>T	3	sp_re&sp_do&int	I14/25	0.002612	1	0	0.00	0.00	0.00	0.00	0.00	0.00	0.00	yes	no
	c.6122T>G	p.I2041R	9:g.132288636A>C	3	mis	E16/26	0.000000	1	0	0.72	0.67	0.44	0.83	0.00	0.00	7.35	no	no
	c.6166G>A	p.V2056I	9:g.132288592C>T	3	mis	E16/26	0.000065	1	0	0.55	0.64	0.28	0.33	0.00	0.00	4.55	yes	no
	c.6313C>T	p.R2105W	9:g.132288247G>A	3	mis	E17/26	0.000279	1	0	0.44	0.75	0.05	0.46	0.00	0.00	0.88	yes	no
	c.6806C>T	p.S2269F	9:g.132278106G>A	3	mis	E21/26	0.000058	1	0	0.58	0.83	0.91	0.92	0.00	0.00	7.96	yes	no
	c.7103C>G	p.P2368R	9:g.132271806G>C	3	mis&sp_re	E24/26	0.000015	1	0	0.34	0.35	-0.37	0.59	0.00	0.00	2.62	yes	no
	c.7195A>G	p.I2399V	9:g.132271714T>C	3	mis	E24/26	0.001249	1	0	0.39	0.65	0.29	0.41	0.00	0.00	4.30	yes	no
	c.7720G>A	p.E2574K	9:g.132264553C>T	3	mis	E26/26	0.000227	1	0.03	0.33	0.75	-0.10	0.30	0.00	0.00	1.35	yes	no
	c.7777G>C	p.V2593L	9:g.132264496C>G	3	mis	E26/26	0.000000	1	0.38	0.25	0.31	-0.67	0.18	0.00	0.00	-0.16	no	no
	c.7910C>T	p.A2637V	9:g.132264363G>A	3	mis	E26/26	0.000000	1	0.03	0.28	0.75	-0.08	0.32	0.00	0.00	2.73	no	no
	c.7972A>G	p.R2658G	9:g.132264301T>C	3	mis	E26/26	0.000100	1	0.44	0.21	0.29	-0.61	0.16	0.00	0.00	-0.58	yes	no
SIGMAR1	c.140G>A	p.R47Q	9:g.34637558C>T	3	mis	E1/4	0.000012	1	0.13	0.57	0.15	-0.91	0.09	0.26	0.06	1.55	yes	no
SPG11	c.32C>T	p.A11V	15:g.44663616G>A	3	mis	E1/40	0.000130	1	0.13	0.47	0.29	-0.81	0.09	0.01	0.00	-0.38	yes	no
	c.130C>T	p.R44W	15:g.44663518G>A	3	mis	E1/40	0.000100	1	0	0.70	0.57	0.28	0.48	1.00	0.94	3.31	yes	no
	c.328A>G	p.N110D	15:g.44660546T>C	3	mis	E2/40	0.000522	1	0.25	0.41	0.26	-0.91	0.08	0.00	0.01	2.41	yes	no
	c.395G>A	p.S132N	15:g.44660479C>T	3	mis	E2/40	0.000778	2	0.09	0.34	0.34	-0.71	0.07	0.00	0.01	1.24	yes	no
	c.664A>G	p.I222V	15:g.44659082T>C	3	mis	E3/40	0.000023	1	0.07	0.44	0.06	-1.02	0.03	0.02	0.02	3.96	yes	no
	c.833A>T	p.N278I	15:g.44657131T>A	3	mis	E4/40	0.006769	1	0	0.35	0.06	-1.06	0.05	0.89	0.39	0.01	yes	no
	c.841G>T	p.V281F	15:g.44657123C>A	3	mis	E4/40	0.000524	1	0.07	0.25	0.08	-0.95	0.20	0.69	0.30	-2.79	yes	no
	c.867C>A	p.F289L	15:g.44657097G>T	3	mis&sp_re	E4/40	0.000018	1	0.01	0.58	0.22	-0.71	0.28	1.00	0.96	5.36	yes	no
	c.953C>G	p.P318R	15:g.44652183G>C	3	mis	E5/40	0.000000	1	0.04	0.36	0.43	-0.55	0.21	0.93	0.64	2.79	yes	no
	c.1126A>G	p.N376D	15:g.44651821T>C	3	mis	E6/40	0.001450	3	0.68	0.32	0.14	-0.99	0.08	0.00	0.00	-0.41	yes	no
	c.1248G>T	p.M416I	15:g.44651699C>A	3	mis	E6/40	0.000389	1	0.79	0.39	0.24	-0.88	0.10	0.00	0.00	2.36	yes	no
	c.1939A>G	p.I647V	15:g.44628797T>C	3	mis	E10/40	0.000523	1	0.19	0.50	0.58	0.12	0.31	0.78	0.26	5.80	yes	no
	c.2075T>C	p.I692T	15:g.44626500A>G	3	mis	E11/40	0.000778	1	0	0.56	0.65	0.54	0.55	1.00	0.97	5.46	yes	no
	c.2121C>A	p.F707L	15:g.44626454G>T	3	mis	E11/40	0.000375	1	0.04	0.57	0.57	0.13	0.52	0.74	0.37	1.92	yes	no
*	c.2305C>T	p.R769C	15:g.44622739G>A	3	mis	E12/40	0.000321	1	0	0.53	0.83	0.84	0.80	1.00	0.99	2.81	yes	no
	c.2512T>A	p.C838S	15:g.44621867A>T	3	mis	E14/40	0.000000	1	0.74	0.28	0.12	-0.92	0.07	0.00	0.00	-0.06	no	no
	c.2669G>A	p.R890H	15:g.44620355C>T	3	mis	E15/40	0.000375	2	0.62	0.22	0.18	-0.98	0.10	0.00	0.00	0.01	yes	no
	c.2774T>C	p.I925T	15:g.44620250A>G	3	mis	E15/40	0.000018	1	0.01	0.42	0.48	0.01	0.49	0.49	0.12	7.33	yes	no
	c.2897G>A	p.R966H	15:g.44615504C>T	3	mis	E16/40	0.000730	1	0.57	0.21	0.50	-0.16	0.30	0.68	0.09	0.00	yes	no
	c.2926C>T	p.P976S	15:g.44615475G>A	3	mis	E16/40	0.000000	1	0.03	0.45	0.52	0.06	0.42	0.58	0.27	6.12	no	no

	c.3095C>T	p.P1032L	15:g.44613480G>A	3	mis	E17/40	0.000140	1	0	0.58	0.24	-0.68	0.23	0.99	0.80	7.68	yes	no
¶	c.3320G>C	p.C1107S	15:g.44608577C>G	3	mis	E19/40	0.026316	3	0.74	0.39	0.03	-1.01	0.10	0.00	0.00	0.86	yes	no
	c.3893-20_3893-9del	-	15:g.44598384_44598395del	3	sp_tr&int	I22/39	0.000088	1	0	0.00	0.00	0.00	0.00	0.00	0.00	0.00	yes	no
	c.3946C>T	p.L1316F	15:g.44598320G>A	3	mis	E23/40	0.000749	1	0	0.48	0.67	0.42	0.48	1.00	0.99	6.66	yes	no
‡	c.3956T>C	p.L1319S	15:g.44598310A>G	3	mis	E23/40	0.000009	1	0	0.44	0.57	0.22	0.73	1.00	1.00	6.22	yes	no
	c.4475C>T	p.T1492I	15:g.44595419G>A	3	mis	E26/40	0.000065	1	0	0.46	0.65	0.24	0.43	1.00	0.94	7.42	yes	no
	c.4689G>T	p.R1563S	15:g.44592385C>A	3	mis	E27/40	0.000000	1	0	0.34	0.24	-0.72	0.25	0.01	0.03	2.18	no	no
	c.4753G>T	p.A1585S	15:g.44589405C>A	3	mis	E28/40	0.000015	1	0.4	0.24	0.29	-0.88	0.08	0.00	0.01	-0.21	yes	no
	c.5059G>A	p.A1687T	15:g.44585698C>T	3	mis	E29/40	0.001196	1	0.18	0.37	0.47	-0.11	0.27	0.99	0.59	1.17	yes	no
	c.5121G>T	p.E1707D	15:g.44585636C>A	3	mis&sp_re	E29/40	0.004733	4	0.01	0.39	0.56	0.05	0.33	0.94	0.62	5.98	yes	yes
	c.5725G>A	p.A1909T	15:g.44583955C>T	3	mis	E30/40	0.000362	1	1	0.25	0.28	-0.88	0.08	0.00	0.00	0.22	yes	no
	c.5839G>A	p.D1947N	15:g.44583841C>T	3	mis	E30/40	0.000213	1	0.43	0.27	0.15	-0.99	0.08	0.20	0.02	-0.36	yes	no
	c.5990T>A	p.L1997Q	15:g.44574918A>T	3	mis	E31/40	0.000029	1	0	0.63	0.64	0.29	0.63	1.00	1.00	8.15	yes	no
	c.6175C>T	p.R2059W	15:g.44573577G>A	3	mis	E32/40	0.000181	1	0	0.26	0.42	-0.34	0.33	0.98	0.58	1.98	yes	no
	c.6278G>A	p.R2093H	15:g.44572748C>T	3	mis	E33/40	0.001092	2	0.3	0.22	0.23	-0.91	0.05	0.02	0.00	-0.15	yes	no
	c.6352C>G	p.L2118V	15:g.44570650G>C	3	mis	E34/40	0.000018	1	0	0.69	0.78	0.66	0.67	1.00	1.00	3.88	yes	no
¶	c.6475G>C	p.V2159L	15:g.44570527C>G	3	mis&sp_re	E34/40	0.000000	1	0.49	0.32	0.16	-0.98	0.06	0.02	0.01	0.72	no	no
	c.6490A>G	p.T2164A	15:g.44569493T>C	3	mis	E35/40	0.000921	1	0	0.69	0.68	0.44	0.57	1.00	0.90	5.85	yes	no
	c.6610C>G	p.L2204V	15:g.44567568G>C	3	mis	E36/40	0.000000	1	0	0.77	0.65	0.37	0.56	1.00	1.00	5.01	no	no
	c.6625C>T	p.R2209C	15:g.44567553G>A	3	mis	E36/40	0.000655	4	0	0.75	0.70	0.41	0.62	1.00	1.00	9.92	yes	no
	c.6632G>A	p.R2211H	15:g.44567546C>T	3	mis	E36/40	0.029605	2	0.21	0.36	0.28	-0.81	0.12	0.14	0.01	2.02	yes	no
	c.6707A>T	p.E2236V	15:g.44567471T>A	3	mis	E36/40	0.000015	2	0	0.67	0.71	0.49	0.73	1.00	1.00	7.80	yes	no
	c.6709G>A	p.A2237T	15:g.44567469C>T	3	mis	E36/40	0.000000	1	0.01	0.53	0.61	0.18	0.33	1.00	0.92	3.73	yes	no
	c.6871T>C	p.C2291R	15:g.44565982A>G	3	mis	E38/40	0.000015	2	0	0.79	0.75	0.63	0.84	1.00	1.00	7.27	yes	no
	c.6878G>A	p.R2293Q	15:g.44565975C>T	3	mis	E38/40	0.000575	1	0.03	0.42	0.59	0.16	0.33	1.00	0.99	2.35	yes	no
‡	c.6907C>G	p.H2303D	15:g.44565946G>C	3	mis	E38/40	0.000024	1	0	0.65	0.66	0.29	0.57	1.00	1.00	9.74	yes	no
*	c.6944A>C	p.N2315T	15:g.44565909T>G	3	mis	E38/40	0.002358	1	0.01	0.50	0.67	0.42	0.58	1.00	1.00	4.98	yes	no
	c.6944A>G	p.N2315S	15:g.44565909T>C	3	mis	E38/40	0.000111	1	0.04	0.47	0.67	0.39	0.50	1.00	1.00	4.98	yes	no
	c.7132T>C	p.F2378L	15:g.44564566A>G	3	mis	E39/40	0.006329	2	0	0.56	0.51	-0.15	0.34	1.00	0.95	4.81	yes	no
	c.7324G>A	p.A2442T	15:g.44563129C>T	3	mis	E40/40	0.000375	1	0	0.46	0.37	-0.21	0.27	0.58	0.28	1.12	yes	no
TAF15	c.7+9C>T	-	17:g.35809585C>T	3	int	I1/15	0.000000	1	0	0.00	0.00	0.00	0.00	0.00	0.00	0.00	no	no
	c.22G>A	p.G8S	17:g.35817730G>A	3	mis	E2/16	0.000214	1	0.81	0.45	0.53	-0.46	0.00	0.00	0.00	0.40	yes	no
	c.48-6T>A	-	17:g.35820018T>A	3	sp_re&sp_tr&int	I2/15	0.000088	1	0	0.00	0.00	0.00	0.00	0.00	0.00	0.00	yes	no
	c.205G>T	p.G69C	17:g.35820352G>T	3	mis	E5/16	0.001179	2	0	0.45	0.38	-0.21	0.00	0.96	0.74	4.48	yes	no
	c.259G>A	p.G87R	17:g.35820406G>A	3	mis	E5/16	0.000023	1	0.01	0.64	0.44	-0.14	0.00	0.89	0.71	6.15	yes	no
	c.346T>G	p.Y116D	17:g.35822695T>G	3	mis	E6/16	0.000024	2	0	0.70	0.46	-0.01	0.00	1.00	0.99	4.67	yes	no
	c.416A>G	p.H139R	17:g.35822765A>G	3	mis	E6/16	0.000000	1	0.35	0.63	0.06	-1.06	0.00	0.27	0.03	3.38	no	no
	c.485-9T>C	-	17:g.35824069T>C	3	sp_tr&int	I6/15	0.000100	1	0	0.00	0.00	0.00	0.00	0.00	0.00	0.00	yes	no
	c.674-7dupC	-	17:g.35836125dup	3	sp_re&sp_tr&int	I9/15	0.000000	1	0	0.00	0.00	0.00	0.00	0.00	0.00	0.00	no	no
	c.1088C>T	p.P363L	17:g.35844158C>T	3	mis&sp_re	E13/16	0.000227	1	0.02	0.75	0.17	-0.73	0.00	0.81	0.25	3.13	yes	yes
	c.1143G>T	p.E381D	17:g.35844334G>T	3	mis	E14/16	0.000018	1	0.04	0.62	0.11	-0.96	0.00	0.06	0.06	0.86	yes	no
	c.1296_1325del	p.SSG432_DRS442delinsS	17:g.35844595_35844624del	3	inf_del	E15/16	0.000000	1	0	0.00	0.00	0.00	0.00	0.00	0.00	0.00	no	no
	c.1332_1358del	p.GGY444_SGG453delinsG	17:g.35844631_35844657del	3	inf_del	E15/16	0.000000	1	0	0.00	0.00	0.00	0.00	0.00	0.00	0.00	no	no
	c.1344_1370del	p.GDR448_YGG457delinsG	17:g.35844643_35844669del	3	inf_del	E15/16	0.000000	1	0	0.00	0.00	0.00	0.00	0.00	0.00	0.00	no	no
	c.1446_1466del	p.DRG482_GGD489delinsD	17:g.35844745_35844765del	3	inf_del	E15/16	0.000000	1	0	0.00	0.00	0.00	0.00	0.00	0.00	0.00	no	no
	c.1539_1541del	p.GG513_GG514delinsG	17:g.35844838_35844840del	3	inf_del	E15/16	0.000000	1	0	0.00	0.00	0.00	0.00	0.00	0.00	0.00	no	no
	c.1668_1691del	p.GGY556_RGG564delinsG	17:g.35844967_35844990del	3	inf_del	E15/16	0.000000	1	0	0.00	0.00	0.00	0.00	0.00	0.00	0.00	no	no
	c.1674_1697del	p.YGG558_GGY566delinsY	17:g.35844973_35844996del	3	inf_del	E15/16	0.000000	1	0	0.00	0.00	0.00	0.00	0.00	0.00	0.00	no	no
	c.1739G>A	p.R580K	17:g.35845038G>A	3	mis&sp_re	E15/16	0.000100	1	0	0.81	0.00	0.00	0.00	0.00	0.00	3.53	yes	yes
VEGFA	c.32_33delinsAA	p.S11K	6:g.43770738_43770739delinsAA	3	mis	E1/8	0.000000	1	0.28	0.00	0.00	0.00	0.00	0.00	0.00	0.00	no	no
	c.962+4A>G	-	6:g.43778922A>G	3	sp_re&sp_do&int	I5/7	0.000000	1	0	0.00	0.00	0.00	0.00	0.00	0.00	0.00	no	no
	c.986A>T	p.K329M	6:g.43780755A>T	3	mis	E6/8	0.000000	1	0.01	0.61	0.06	-1.06	0.00	1.00	0.99	6.74	no	no
	c.1003C>T	p.R335C	6:g.43780772C>T	3	mis	E6/8	0.000182	2	0.01	0.80	0.73	0.00	0.00	1.00	0.99	5.74	yes	no
	c.1015C>T	p.R339W	6:g.43780784C>T	3	mis	E6/8	0.001092	1	0	0.72	0.75	0.23	0.00	0.00	0.00	5.53	yes	no
	c.1118A>G	p.H373R	6:g.43781988A>G	3	mis	E7/8	0.000700	1	0.24	0.74	0.09	-0.97	0.15	0.00	0.00	7.20	yes	no
	c.1160A>G	p.K387R	6:g.43782030A>G	3	mis	E7/8	0.000163	1	0.2	0.70	0.07	-1.08	0.11	0.00	0.00	7.17	yes	no
VPS54	c.109C>T	p.P37S	2:g.63983891G>A	3	mis	E2/23	0.000000	1	0	0.73	0.09	-0.72	0.30	1.00	1.00	8.58	no	no
	c.374C>G	p.S125C	2:g.63981650G>C	3	mis	E3/23	0.000026	1	0.04	0.47	0.06	-1.02	0.05	0.64	0.26	3.67	yes	no
	c.452C>T	p.T151I	2:g.63972171G>A	3	mis	E4/23	0.000000	1	0.21	0.66	0.05	-1.04	0.02	0.00	0.00	2.10	yes	no
	c.455A>T	p.H152L	2:g.63972168T>A	3	mis&sp_re	E4/23	0.000117	2	0.66	0.76	0.05	-1.08	0.10	0.04	0.01	4.98	yes	no
	c.643A>G	p.I215V	2:g.63962425T>C	3	mis	E7/23	0.000000	1	0.62	0.50	0.04	-1.07	0.07	0.01	0.01	5.79	no	no
	c.1103G>A	p.R368K	2:g.63949071C>T	3	mis	E8/23	0.000024	1	0.08	0.58	0.09	-1.04	0.17	0.63	0.22	6.52	yes	no
	c.1906A>T	p.I636L	2:g.63920591T>A	3	mis	E14/23	0.000029	1	0.45	0.50	0.02	-1.00	0.12	0.00	0.00	4.83	yes	no

c.2164+6T>C	-	2:g.63919877A>G	3	sp_re&sp_do&int	I15/22	0.000000	1	0	0.00	0.00	0.00	0.00	0.00	0.00	0.00	no	no
c.2198T>C	p.I733T	2:g.63916930A>G	3	mis	E16/23	0.000523	1	0.17	0.43	0.04	-1.02	0.05	0.00	0.01	3.72	yes	no
c.2335-10C>A	-	2:g.63913320G>T	3	sp_tr&int	I17/22	0.000008	1	0	0.00	0.00	0.00	0.00	0.00	0.00	0.00	yes	no
c.2603T>C	p.L868S	2:g.63912367A>G	3	mis	E20/23	0.000100	2	0.15	0.83	0.09	-1.07	0.34	0.14	0.05	9.33	yes	no
c.2695G>A	p.E899K	2:g.63899512C>T	3	mis	E21/23	0.000200	1	0	0.78	0.17	-0.92	0.31	1.00	0.97	7.27	yes	no
